# Supplementary material for: Effect of communicating community immunity on COVID-19 vaccine-hesitant people from ethnically diverse backgrounds: an experimental vignette study in the UK
Source: BMJ Open. 2022 Nov 3;12(11):e065804. doi: 10.1136/bmjopen-2022-065804 (PMC9638751; doi:10.1136/bmjopen-2022-065804)
Supplement: Supplementary data [file bmjopen-2022-065804supp001.pdf]

Page 1:

Title of Project: The effect of communicating herd immunity on vaccination intention

This study has been approved by xxxx

We would like to invite you to take part in this study. You should only take part if you want to. Before you decide whether to take part, it is important for you to read the following information carefully and discuss it with others if you wish. Please ask us if anything is unclear or if you would like more information.

What is the purpose of the study?
This study is concerned with how individuals make choices related to the coronavirus vaccination. To explore this further, we have developed a survey to ask people about their preferences.

Why have I been invited?

We are inviting adults (men and women aged 18-65) living in England to take part in this survey.

Do I have to take part?
It is up to you to decide to join the study. If you decide to withdraw you will be asked what you wish to happen to the data you have provided up that point.

What will I have to do if I take part?
If you choose to take part we would ask you to complete an online survey which should take around 10 minutes to complete.

What are the possible disadvantages and risks of taking part?
There are no physical risks for the participants. This study carries very little risk for the participants. There are no right or wrong answers to the questions. As we are asking you about health issues that may be sensitive, there is a slight possibility that you will feel uncomfortable.

What are the possible benefits of taking part?
In addition to the remuneration you receive from Qualtrics, this study will help us to better understand people's attitudes towards coronavirus vaccine.

What if there is a problem?
If you have a concern about any aspects of the study you should in the first instance contact XXX who will do her best to answer your questions. If you would like to discuss a problem with someone not directly involved in the project, you can contact the chairs of xxx research ethics committees (XXX).

Will my taking part in this project be kept confidential?
Yes. All information which is collected about you during the course of the research is anonymous and not linked to any of your personal details. This means that you cannot be recognised from the information you provide. Our procedures for storage, processing, handling and destroying the information you give us are in line with Data

Protection Act (1998) and the General Data Protection Regulation (2016). The survey will be conducted using SurveyMonkey which stores data collected on servers located in the United States.

What will happen to the results of the research project?
We hope to report the findings from the study in an academic journal. You will not be identified in any reports or publications from the study.

Who is organising the research?
This work is being delivered by the National Institute for Health Research Policy Research Unit in Behavioural Science. The Unit is a collaboration between Newcastle University, University College London, University of Warwick, and the London School of Hygiene and Tropical Medicine. The work was developed in collaboration with Public Health England.

Contact for further information
Researcher: XXX
Principal Researcher: XXX
UCL Data Protection Officer: XXX

Thank you for reading this information sheet and for considering to take part in this research study.

[continue page 2]

Consent form

Thank you for considering taking part in this research. If you have any questions arising from the Information explanation already given to you, please ask the researcher before you decide whether to join in.

I confirm that I understand that by ticking/initialling each box below I am consenting to this element of the study. I understand that it will be assumed that unticked/uninitialled boxes means that I DO NOT consent to that part of the study. I understand that by not giving consent for any one element that I may be deemed ineligible for the study.

	**Tick Box**
**1. I confirm that I have read and understood the information sheet for the study above. I have had an opportunity to consider the information and what will be expected of me. I have also had the opportunity to ask questions.**	
**2. I understand that my participation is voluntary and that I am free to withdraw at any time without giving a reason**	
**3. I understand that my data gathered in this study will be stored anonymously and securely. It will not be possible to identify me in any publications.**	
**4. I understand that the data will not be made available to any commercial organisations but is solely the responsibility of the researchers undertaking this study.**	
**5. I am aware of who I should contact if I wish to lodge a complaint.**	
**6. I voluntarily agree to take part in this study.**	
**7. I understand that information collected in this survey is stored on servers based in the USA.**	

[continue page 3]

Page 3: First, we want to make sure you're a good fit for our study. Please answer the questions below so we can check.

1. How old are you in years?
   - 17 or younger [filter out]
   - 18-24 years old
   - 25-34 years old
   - 35-44 years old
   - 55-54 years old
   - 55 or older

2. What is your gender?
   - Female
   - Male
   - Other

3. Where do you live?

   - Scotland [filter out]
   - Northern Ireland [filter out]
   - Wales [filter out]
   - North East
   - North West
   - Yorkshire and the Humber
   - West Midlands
   - East Midlands
   - South West
   - South East
   - East of England
   - Greater London

4. Which of the following describes your ethnic group?
   - White British [filter out]
   - Other White background [filter out]
   - Black background
   - Asian background
   - Mixed background
   - Any other

5. Have you already been invited for a coronavirus vaccine?
   - Yes [filter out]
   - No

6. Do you or someone in your household have a health condition, is pregnant or age 70+? (question adapted from NIHR questionnaire 2020)
   - Yes
   - No

7. Which of the following numbers represents the biggest risk of getting a disease? (question from Lipkus et al., 2001, used in Stoffel et al., 2019)

- 1/10
- 1/100
- 1/1000
- Don't know

[continue page 4]

Page 4:
Thank you for filling out the initial questions. We would now like to introduce the main part of the survey.

The NHS is offering free coronavirus vaccine to people living in England.

8. Please evaluate how much you disagree or agree with the following statement.

When it's available to me, I will have a coronavirus vaccine. (question adapted from YouGov questionnaire)

- Strongly disagree [continue page 5/6/7]
- Disagree [continue page 5/6/7]
- Neither agree nor disagree [continue page 5/6/7]
- Agree [filter out]
- Strongly agree [filter out]

Page 5: Control condition (33.3%):

Imagine that you have received the following invitation letter from the NHS.

NHS | HM Government

CORONAVIRUS VACCINATION

We are writing to invite you to get a free coronavirus vaccine.

This is part of a national programme that will involve millions of people receiving this vaccine. Each person who gets the vaccine will need to receive two separate doses.

In order to get the vaccine, you will need to go to your registered GP. The two doses will be given to you on two separate visits

Get vaccinated to protect you from Covid-19.

9a. 1. Would you get vaccinated against the coronavirus? (1 means definitely not while 8 indicates definitely yes) (question adapted from unpublished study by Stoffel & Herrmann)

- 1
- 2
- 3
- 4
- 5
- 6
- 7
- 8

10a. Do you think that most people will get vaccinated? (question adapted from unpublished study by Stoffel & Herrmann)

- No
- Yes

11a. Do you believe that getting vaccinated against the virus is socially important? (question adapted from unpublished study by Stoffel & Herrmann)

- No
- Yes

[continue page 8]

Page 6: Society condition (33.3%):

Imagine that you have received the following invitation letter from the NHS.

NHS | HM Government

CORONAVIRUS VACCINATION

We are writing to invite you to get a free coronavirus vaccine.

This is part of a national programme that will involve millions of people receiving this vaccine. Each person who gets the vaccine will need to receive two separate doses.

In order to get the vaccine, you will need to go to your registered GP. The two doses will be given to you on two separate visits

Get vaccinated to protect you and the vulnerable people in Great Britain from Covid-19.

9b. 1. Would you get vaccinated against the coronavirus? (1 means definitely not while 8 indicates definitely yes) (question adapted from unpublished study by Stoffel & Herrmann)

- 1
- 2
- 3
- 4
- 5
- 6
- 7
- 8

10b. Do you think that most people will get vaccinated? (question adapted from unpublished study by Stoffel & Herrmann)

- No
- Yes

11b. Do you believe that getting vaccinated against the virus is socially important? (question adapted from unpublished study by Stoffel & Herrmann)

- No
- Yes

[continue page 8]

Page 7: Friends condition (33.3%):

Imagine that you have received the following invitation letter from the NHS.

NHS | HM Government

CORONAVIRUS VACCINATION

We are writing to invite you to get a free coronavirus vaccine.

This is part of a national programme that will involve millions of people receiving this vaccine. Each person who gets the vaccine will need to receive two separate doses.

In order to get the vaccine, you will need to go to your registered GP. The two doses will be given to you on two separate visits

Get vaccinated to protect you and your vulnerable friends and family members, such as your children, your parents or your grandparents from Covid-19.

9c. 1. Would you get vaccinated against the coronavirus? (1 means definitely not while 8 indicates definitely yes) (question adapted from unpublished study by Stoffel & Herrmann)

- 1
- 2
- 3
- 4
- 5
- 6
- 7
- 8

10c. Do you think that most people will get vaccinated? (question adapted from unpublished study by Stoffel & Herrmann)

- No
- Yes

11c. Do you believe that getting vaccinated against the virus is socially important? (question adapted from unpublished study by Stoffel & Herrmann)

- No
- Yes

[continue page 8]

Page 8: Attitudes towards vaccination (taken from Betsch et al., 2018)

12. Please indicate how strongly you agree or disagree with all the following statements

Item	Response options						
	**strongly disagree**	**moderately disagree**	**slightly disagree**	**neutral**	**slightly agree**	**moderately agree**	**Strongly agree**
I am completely confident that vaccines are safe.							
Vaccinations are effective.							
Regarding vaccines, I am confident that public authorities decide in the best interest of the community.							
Everyday stress prevents me from getting vaccinated.							
For me, it is inconvenient to receive vaccinations.							
Visiting the doctor's makes me feel uncomfortable; this keeps me from getting vaccinated.							
When I think about getting vaccinated, I weigh benefits and risks to make the best decision possible.							
For each and every vaccination, I closely consider whether it is useful for me.							
It is important for me to fully understand the topic of vaccination, before I get vaccinated.							

[continue page 8]

Page 9. Additional information

13. Before continuing with the survey, would you be interested in reading some more facts and figures about the vaccination appointment on the next page or do you prefer to skip it? (question adapted from Stoffel et al., 2019)

- Read information on next page before continuing with survey [continue page 10]
- Skip information on next page and continue with survey [continue page 12]

Page 10: Information about the vaccination process.

How the coronavirus vaccine is given

The coronavirus vaccine should be administered by intramuscular injection into the deltoid muscle of the upper arm.

It's given as 2 doses. You will have the 2nd dose 3 to 12 weeks after having the 1st dose.

How safe is the coronavirus vaccine?

The vaccines approved for use in the UK have met strict standards of safety, quality and effectiveness set out by the independent Medicines and Healthcare products Regulatory Agency (MHRA).

Any coronavirus vaccine that is approved must go through all the clinical trials and safety checks all other licensed medicines go through. The MHRA follows international standards of safety.

Other vaccines are being developed. They will only be available on the NHS once they have been thoroughly tested to make sure they are safe and effective.

So far, thousands of people have been given a coronavirus vaccine and reports of serious side effects, such as allergic reactions, have been very rare. No long-term complications have been reported.

Coronavirus vaccine side effects

Most side effects of the coronavirus vaccine, if you do experience any, are mild and should not last longer than a week, such as:

- a sore arm where the needle went in
- feeling tired
- a headache
- feeling achy
- feeling or being sick

You can take painkillers, such as paracetamol, if you need to.

If you have a high temperature you may have coronavirus or another infection.

As with any symptoms, if they get worse or you are worried, call 111.

Allergic reactions

Tell healthcare staff before you are vaccinated if you've ever had an unexplained anaphylaxis or a serious allergic reaction (anaphylaxis) due to food, an identified drug or vaccine, multiple classes of drugs, or an insect sting.

You should not have the vaccine if you've ever had a serious allergic reaction to:

- a previous vaccine
- a previous dose of the same COVID-19 vaccine
- some medicines, household products or cosmetics (especially those which contain polyethylene glycol (PEG) or any of the other ingredients of the vaccine(s) (PHE, 2021))

Serious allergic reactions are very rare. If you do have a reaction to the vaccine, it usually happens in minutes. Staff giving the vaccine are trained to deal with allergic reactions and treat them immediately.

[continue page 11]

Page 11: Comprehension questions on the additional information

14a. When would you be given the second dose of the vaccine
- 1-2 weeks after the first one
- 3-12 weeks after the first one
- 13-20 weeks after the first one

14b. Is high temperature a common side effect of the vaccine?
- Yes
- No

14c. Are you allowed to take painkillers in case you suffer from side effects?
- Yes
- No

14d. When would allergic reaction to the vaccine usually happen?
- In seconds
- In minutes
- In hours

[continue page 12]

Page 12: About yourself (question adapted from Stoffel et al., 2019)

15. What is the highest educational qualification you have?
- no qualifications
- GCSE level
- A-level
- higher education

16. Are you currently in paid employment?
- Yes
- No

17. What is your marital status?
- Single
- Married or living with partner
- Divorced, separated or widowed

[continue page 13]

Page 13: End of the survey

Thank you for your participation!
The information collected from this survey will be used to examine attitudes towards coronavirus vaccination. The aim of this study is to investigate the role of the appointment characteristics on vaccination intentions among adults (men and women aged 18-50) with low interest. If you would like more information about the study, please contact XXX.

[redirect to the survey vendors website according to provided link]

References

Betsch, C., Schmid, P., Heinemeier, D., Korn, L., Holtmann, C., & Böhm, R. (2018). Beyond confidence: Development of a measure assessing the 5C psychological antecedents of vaccination. PloS one, 13(12), e0208601. https://doi.org/10.1371/journal.pone.0208601

Lipkus I.M., Samsa G., Rimer B.K. (2001). General performance on a numeracy scale among highly educated samples. Medical Decision Making, 21(1), 37–44. https://doi.org/10.1177/0272989X0102100105 PMID: 11206945

Public Health England (PHE). (2021) COVID-19: the green book, chapter 14a (*Last updated 26 January 2021*). *Available at PHE gateway number 2020300. https://www.gov.uk/government/publications/covid-19-the-green-book-chapter-14a*

Stoffel, S., & Herrmann, B. (2021). How Does Communicating Herd Immunity Affect Immunization Intentions?: Evidence From a Cross-Country Online Experiment. International Journal of Applied Behavioral Economics (IJABE), 10(4), 12-20.

Stoffel, S. T., Yang, J., Vlaev, I., & von Wagner, C. (2019). Testing the decoy effect to increase interest in colorectal cancer screening. PloS one, 14(3), e0213668.
