# Supplementary material for: Effect of communicating community immunity on COVID-19 vaccine-hesitant people from ethnically diverse backgrounds: an experimental vignette study in the UK
Source: BMJ Open. 2022 Nov 3;12(11):e065804. doi: 10.1136/bmjopen-2022-065804 (PMC9638751; doi:10.1136/bmjopen-2022-065804)
Supplement: Supplementary data [file bmjopen-2022-065804supp002.pdf]

Supplementary tables

Table S1: Reasons for not wanting to get vaccinated after experimental manipulation*

	Overall sample (N=306)		Only White ethnic background (N=118)		Model 3: Black, Asian, mixed or other ethnic background (N=188)	
Reason	N	(%)	N	(%)	N	(%)
Fear of side effect	201	65.9	83	70.9	118	62.8
Vaccine does not offer protection	115	37.7	47	40.2	68	36.2
Critical reports in media	69	22.6	25	21.4	44	23.4
General objections against vaccination	63	20.7	28	23.9	35	18.6
Fear of needles	44	14.4	20	17.1	24	12.8
Other reasons	37	12.1	16	13.7	21	11.2
Disease is harmless	30	9.8	8	6.8	22	11.7
Discouraged by family/friends' opinion	26	8.5	9	7.7	17	9.0
Discouraged by physician's opinion	17	5.6	9	7.7	8	4.3
Difficulties getting to the appointment	14	4.6	6	5.1	8	4.3
Too much effort	8	2.6	2	1.7	6	3.2

*only answered by study participants that stated low vaccination intentions [1-4] after the experimental manipulation

Table S2: Descriptive statistics across experimental conditions

	Control (N=167)		Society (N=176)		Family and friends (N=169)		Total (N=512)	
	N	(%)	N	(%)	N	(%)	N	(%)
Vaccination status								
Have been invited	101	(60.5%)	94	(53.4%)	107	(63.3%)	302	(59.0%)
Not been invited yet	66	(39.5%)	82	(46.6%)	62	(36.7%)	210	(41.0%)
Age								
18-24 years old	54	(32.3%)	52	(29.5%)	43	(25.4%)	149	(29.1%)
25-34 years old	74	(44.3%)	90	(51.1%)	72	(42.6%)	236	(46.1%)
35-44 years old	14	(8.4%)	14	(8.0%)	35	(20.7%)	63	(12.3%)
45+ years old	25	(15.0%)	20	(11.3%)	19	(11.2%)	64	(12.5%)
Gender								
Male	74	(44.3%)	81	(46.0%)	67	(39.6%)	222	(43.4%)
Female	93	(55.7%)	93	(52.8%)	99	(58.6%)	285	(55.7%)
Non-binary	0	(0.0%)	2	(1.1%)	3	(1.8%)	5	(1.0%)
Ethnicity								
White	72	(43.1%)	78	(44.3%)	57	(33.7%)	207	(40.4%)
Mixed	16	(9.6%)	30	(17.0%)	23	(13.6%)	69	(13.5%)
Asian	30	(18.0%)	34	(19.3%)	36	(21.3%)	100	(19.5%)
Black	45	(26.9%)	32	(18.2%)	46	(27.2%)	123	(24.0%)
Arab or other	4	(2.4%)	2	(1.1%)	7	(4.1%)	13	(2.5%)
A-levels								
No	29	(17.4%)	31	(17.6%)	46	(27.2%)	106	(20.7%)
Yes	138	(82.6%)	145	(82.4%)	123	(72.8%)	406	(79.3%)
Paid employment								
No	49	(29.3%)	47	(26.7%)	58	(34.3%)	154	(30.1%)
Yes	118	(70.7%)	129	(73.3%)	111	(65.7%)	358	(69.9%)
Living condition								
Alone	89	(53.3%)	91	(51.7%)	96	(56.8%)	276	(53.9%)
With someone	78	(46.7%)	85	(48.3%)	73	(43.2%)	236	(46.1%)

Table S3: Binary logistic regression on active interest to read more about vaccination

		Model 1: Overall sample					Model 2: Only White ethnic background					Model 3: Black, Asian, mixed or other ethnic backgound			
	(%)	OR	95% CI	aOR	95% CI	(%)	OR	95% CI	aOR	95% CI	(%)	OR	95% CI	aOR	95% CI
Condition															
Control	34.7	Ref.		Ref.		33.3	Ref.		Ref.		35.8	Ref.		Ref.	
Society	44.9	1.531	0.990 - 2.366	1.472	0.940 - 2.307	42.3	1.446	0.726 - 2.882	1.587	0.891 - 2.827	46.9	1.587	0.891 - 2.827	1.570	0.870 - 2.833
Family and friends	42.6	1.395	0.898 - 2.168	1.484	0.933 - 2.360	47.4	1.896	0.876 - 4.103	1.205	0.685 - 2.119	40.1	1.205	0.685 - 2.119	1.270	0.704 - 2.290
Vaccination status															
Invited	38.1	Ref.		Ref.		38.6	Ref.		Ref.		37.7	Ref.		Ref.	
Not invited	44.8	1.318	0.921 - 1.884	1.581	1.067 - 2.342*	42.8	1.238	0.701 - 2.185	1.456	0.770 - 2.754	45.4	1.372	0.865 - 2.176	1.621	0.979 - 2.685
Age															
18-24 years old	36.9	Ref.		Ref.		33.3	Ref.		Ref.		38.6	Ref.		Ref.	
25-34 years old	39.4	1.112	0.728 - 1.697	1.182	0.735 - 1.901	40.2	1.343	0.664 - 2.717	1.597	0.734 - 3.474	38.7	1.002	0.581 - 1.727	0.901	0.489 - 1.662
35-44 years old	50.8	1.764	0.973 - 3.200	1.722	0.889 - 3.333	53.8	2.333	0.672 - 8.101	2.726	0.707 - 10.503	50.0	1.590	0.802 - 3.150	1.501	0.693 - 3.252
45+ years old	45.3	1.416	0.782 - 2.565	1.915	0.986 - 3.718	48.3	1.867	0.726 - 4.796	2.415	0.839 - 6.951	42.9	1.192	0.546 - 2.601	1.351	0.559 - 3.266
Gender															
Male	42.3	Ref.		Ref.		39.8	Ref.		Ref.		44.5	Ref.		Ref.	
Female	39.6	0.895	0.626 - 1.278	0.898	0.617 - 1.308	41.6	1.076	0.616 - 1.882	1.113	0.614 - 2.019	38.6	0.782	0.490 - 1.249	0.809	0.494 - 1.325
Non-binary	40.0	0.908	0.149 - 5.541	0.657	0.102 - 4.213	33.3	0.756	0.066 - 8.611	0.547	0.045 - 6.582	50.0	1.245	0.076 - 20.382	1.112	0.065 - 19.070
Ethnicity															
White	40.6	Ref.		Ref.					.			.		.	
Mixed	40.6	1.000	0.574 - 1.742	0.908	0.510 - 1.616										
Asian	52.0	1.586	0.981 - 2.565	1.468	0.884 - 2.440										
Black	34.9	0.787	0.495 - 1.250	0.742	0.456 - 1.209										
Arab or other	15.4	0.266	0.058 - 1.232	0.280	0.059 - 1.323										
A-levels															
No	33.0	Ref.		Ref.		32.7	Ref.		Ref.		33.3	Ref.		Ref.	
Yes	42.9	1.521	0.970 - 2.386	1.692	1.042 - 2.746*	43.2	1.568	0.809 - 3.035	2.253	1.072 - 4.735*	42.6	1.486	0.801 - 2.759	1.611	0.827 - 3.140
Living condition															
Alone	39.1	Ref.		Ref.		41.1	Ref.		Ref.		38.1	Ref.		Ref.	
With somebody	42.8	1.164	0.817 - 1.657	0.973	0.652 - 1.452	40.2	0.964	0.553 - 1.683	0.873	0.476 - 1.603	45.2	1.337	0.841 - 2.125	1.196	0.700 - 2.041
Paid employment															
No	38.3	Ref.		Ref.		49.1	Ref.		Ref.		32.0	Ref.		Ref.	
Yes	41.9	1.161	0.789 - 1.710	1.107	0.727 - 1.686	37.3	0.617	0.333 - 1.142	0.606	0.310 - 1.183	45.5	1.756	1.058 - 2.914*	1.627	0.947 - 2.795
N		512		512			207		207			305		305	

* *p*<0.05; ** *p*<0.01

Table S4: Ordered logistic regression on perceived importance of vaccination [1;5]

	Model 1: Overall sample				Model 2: Only White ethnic background				Model 3: Black, Asian, mixed or other ethnic backgound			
	OR	95% CI	aOR	95% CI	OR	95% CI	aOR	95% CI	OR	95% CI	aOR	95% CI
Condition												
Control	Ref.		Ref.		Ref.		Ref.		Ref.		Ref.	
Society	0.948	0.649 - 1.386	0.897	0.608 - 1.325	1.493	0.836 - 2.668	1.458	0.794 - 2.677	0.647	0.391 - 1.072	0.627	0.376 - 1.047
Family and friends	0.931	0.639 - 1.357	1.042	0.706 - 1.539	1.271	0.692 - 2.334	1.833	0.961 - 3.496	0.751	0.462 - 1.222	0.829	0.502 - 1.369
Vaccination status												
Invited	Ref.		Ref.		Ref.		Ref.		Ref.		Ref.	
Not invited	1.317	0.964 - 1.799	1.200	0.859 - 1.677	0.919	0.562 - 1.503	0.563	0.321 - 0.990*	1.728	1.149 - 2.599**	1.774	1.151 - 2.733**
Age												
18-24 years old	Ref.		Ref.		Ref.		Ref.		Ref.		Ref.	
25-34 years old	0.851	0.592 - 1.224	0.813	0.547 - 1.208	0.953	0.527 - 1.724	0.754	0.397 - 1.432	0.703	0.439 - 1.126	0.690	0.411 - 1.159
35-44 years old	0.754	0.451 - 1.260	0.689	0.393 - 1.208	0.495	0.180 - 1.362	0.364	0.119 - 1.112	0.905	0.491 - 1.669	0.905	0.463 - 1.769
45+ years old	0.401	0.238 - 0.675**	0.428	0.240 - 0.761**	0.321	0.143 - 0.719**	0.265	0.105 - 0.665**	0.478	0.237 - 0.965*	0.501	0.230 - 1.094
Gender												
Male	Ref.		Ref.		Ref.		Ref.		Ref.		Ref.	
Female	0.557	0.405 - 0.765**	0.537	0.387 - 0.745**	0.411	0.249 - 0.680**	0.375	0.223 - 0.631**	0.715	0.472 - 1.085	0.707	0.459 - 1.090
Non-binary	0.552	0.130 - 2.341	0.509	0.118 - 2.205	0.857	0.154 - 4.754	1.124	0.182 - 6.955	0.163	0.014 - 1.941	0.153	0.012 - 1.891
Ethnicity												
White	Ref.		Ref.				.		.		.	
Mixed	0.701	0.433 - 1.137	0.684	0.416 - 1.125								
Asian	1.132	0.740 - 1.731	1.100	0.707 - 1.710								
Black	0.718	0.482 - 1.071	0.633	0.417 - 0.960*								
Arab or other	0.693	0.259 - 1.854	0.707	0.253 - 1.975								
A-levels												
No	Ref.		Ref.		Ref.		Ref.		Ref.		Ref.	
Yes	1.741	1.188 - 2.549**	1.793	1.190 - 2.701**	2.221	1.277 - 3.865**	2.301	1.260 - 4.204**	1.435	0.840 - 2.453	1.466	0.827 - 2.599
Living condition												
Alone	Ref.		Ref.		Ref.		Ref.		Ref.		Ref.	
With somebody	1.036	0.758 - 1.416	1.088	0.770 - 1.536	0.908	0.558 - 1.478	0.811	0.483 - 1.360	1.117	0.742 - 1.683	1.415	0.883 - 2.266
Paid employment												
No	Ref.		Ref.		Ref.		Ref.		Ref.		Ref.	
Yes	1.263	0.906 - 1.761	1.120	0.788 - 1.590	1.545	0.910 - 2.625	1.252	0.714 - 2.196	1.093	0.711 - 1.679	1.076	0.680 - 1.702
N	512		512		207		207		305		305	

* p<0.05; ** p<0.01

Table S5: Ordered logistic regression on expected uptake of vaccination [1;5]

	Model 1: Overall sample				Model 2: Only White ethnic background				Model 3: Black, Asian, mixed or other ethnic backgound			
	OR	95% CI	aOR	95% CI	OR	95% CI	aOR	95% CI	OR	95% CI	aOR	95% CI
Condition												
Control	Ref.		Ref.		Ref.		Ref.		Ref.		Ref.	
Society	1.036	0.696 - 1.540	1.048	0.700 - 1.571	1.260	0.694 - 2.288	1.443	0.779 - 2.674	0.856	0.501 - 1.461	0.863	0.504 - 1.480
Family and friends	0.864	0.585 - 1.276	0.885	0.592 - 1.324	0.852	0.443 - 1.636	1.000	0.508 - 1.970	0.874	0.535 - 1.429	0.823	0.496 - 1.365
Vaccination status												
Invited	Ref.		Ref.		Ref.		Ref.		Ref.		Ref.	
Not invited	0.598	0.431 - 0.830**	0.640	0.451 - 0.908*	0.491	0.290 - 0.831**	0.513	0.290 - 0.909*	0.675	0.443 - 1.029	0.720	0.459 - 1.129
Age												
18-24 years old	Ref.		Ref.		Ref.		Ref.		Ref.		Ref.	
25-34 years old	1.118	0.765 - 1.635	1.017	0.666 - 1.553	1.375	0.734 - 2.574	1.106	0.562 - 2.179	0.915	0.561 - 1.493	0.988	0.573 - 1.702
35-44 years old	1.405	0.818 - 2.415	1.324	0.737 - 2.380	1.283	0.399 - 4.120	1.431	0.444 - 4.608	1.442	0.778 - 2.673	1.486	0.748 - 2.954
45+ years old	1.472	0.849 - 2.553	1.332	0.727 - 2.441	1.479	0.622 - 3.517	1.197	0.464 - 3.088	1.463	0.708 - 3.020	1.557	0.694 - 3.493
Gender												
Male	Ref.		Ref.		Ref.		Ref.		Ref.		Ref.	
Female	0.839	0.606 - 1.162	0.802	0.573 - 1.123	0.840	0.503 - 1.402	0.838	0.495 - 1.418	0.875	0.570 - 1.345	0.830	0.533 - 1.292
Non-binary	0.256	0.056 - 1.171	0.289	0.060 - 1.401	0.170	0.021 - 1.342	0.231	0.029 - 1.867	0.406	0.048 - 3.450	0.469	0.050 - 4.374
Ethnicity												
White	Ref.		Ref.				.		.		.	
Mixed	0.839	0.507 - 1.390	0.842	0.502 - 1.412								
Asian	1.067	0.686 - 1.659	1.035	0.649 - 1.650								
Black	0.653	0.432 - 0.989*	0.653	0.425 - 1.005								
Arab or other	0.838	0.311 - 2.258	0.896	0.324 - 2.480								
A-levels												
No	Ref.		Ref.		Ref.		Ref.		Ref.		Ref.	
Yes	1.312	0.873 - 1.973	1.272	0.827 - 1.958	1.408	0.771 - 2.571	1.249	0.661 - 2.362	1.306	0.744 - 2.292	1.363	0.741 - 2.505
Living condition												
Alone	Ref.		Ref.		Ref.		Ref.		Ref.		Ref.	
With somebody	0.995	0.722 - 1.370	0.828	0.581 - 1.179	1.019	0.612 - 1.696	0.858	0.501 - 1.469	0.942	0.620 - 1.432	0.852	0.533 - 1.362
Paid employment												
No	Ref.		Ref.		Ref.		Ref.		Ref.		Ref.	
Yes	1.158	0.815 - 1.644	1.102	0.761 - 1.596	1.686	0.950 - 2.990	1.555	0.859 - 2.812	0.914	0.585 - 1.427	0.876	0.545 - 1.408
N	512		512		207		207		305		305	

* p<0.05; ** p<0.01

Table S6. Adjusted logistic regression with interaction terms for condition and ethnicity on intentions, expectations and perceived social importance and active interest (N=512)

	Model 1: Intentions [1;8]		Model 2: Expectations [1;5]		Model 3: Social importance [1;5]		Model 4: Active interest [0;1]	
	aOR	95% CI	aOR	95% CI	aOR	95% CI	aOR	95% CI
Condition and ethnicity								
Control – White	Ref.		Ref.		Ref.		Ref.	
Control – Ethnic minoties	1.304	0.747 - 2.277	1.019	0.570 - 1.824	1.264	0.721 - 2.215	1.041	0.536 - 2.020
Society – White	1.984	1.090 - 3.614*	1.411	0.776 - 2.567	1.529	0.826 - 2.829	1.440	0.730 - 2.839
Society – Ethnic minorities	1.301	0.748 - 2.265	0.873	0.485 - 1.574	0.842	0.480 - 1.476	1.661	0.870 - 3.172
Family and friends - White	1.681	0.886 - 3.189	0.964	0.501 - 1.857	1.568	0.825 - 2.981	1.931	0.921 - 4.047
Family and friends – Ethnic minority	1.419	0.818 - 2.462	0.836	0.476 - 1.465	1.026	0.589 - 1.787	1.287	0.673 - 2.460
Vaccination status								
Invited	Ref.		Ref.		Ref.		Ref.	
Not invited	1.135	0.819 - 1.574	0.625	0.440 - 0.887**	1.164	0.833 - 1.627	1.515	1.026 - 2.237*
Age								
18-24 years old	Ref.		Ref.		Ref.		Ref.	
25-34 years old	0.951	0.647 - 1.397	1.025	0.674 - 1.559	0.804	0.543 - 1.191	1.152	0.721 - 1.840
35-44 years old	0.844	0.490 - 1.454	1.404	0.784 - 2.514	0.749	0.431 - 1.302	1.918	0.999 - 3.682
45+ years old	0.293	0.164 - 0.523**	1.346	0.737 - 2.460	0.428	0.241 - 0.761**	1.824	0.943 - 3.526
Gender								
Male	Ref.		Ref.		Ref.		Ref.	
Female	0.677	0.491 - 0.933*	0.829	0.593 - 1.158	0.546	0.394 - 0.757**	0.908	0.626 - 1.317
Non-binary	0.360	0.082 - 1.577	0.281	0.059 - 1.340	0.457	0.107 - 1.951	0.715	0.112 - 4.552
A-levels								
No	Ref.		Ref.		Ref.		Ref.	
Yes	1.629	1.079 - 2.458*	1.264	0.820 - 1.948	1.822	1.210 - 2.745**	1.783	1.097 - 2.899*
Living condition								
Alone	Ref.		Ref.		Ref.		Ref.	
With somebody	1.263	0.902 - 1.768	0.856	0.602 - 1.216	1.127	0.800 - 1.588	1.012	0.682 - 1.503
Paid employment								
No	Ref.		Ref.		Ref.		Ref.	
Yes	1.145	0.809 - 1.619	1.088	0.753 - 1.572	1.112	0.784 - 1.578	1.101	0.728 - 1.666
N	512		512		512		512	

* *p*<0.05; ** *p*<0.01
