# Supplementary material for: Effect of communicating community immunity on COVID-19 vaccine-hesitant people from ethnically diverse backgrounds: an experimental vignette study in the UK
Source: BMJ Open. 2022 Nov 3;12(11):e065804. doi: 10.1136/bmjopen-2022-065804 (PMC9638751; doi:10.1136/bmjopen-2022-065804)

Supplementary figures

Figure S1. Distribution of vaccination intentions across the experimental conditions

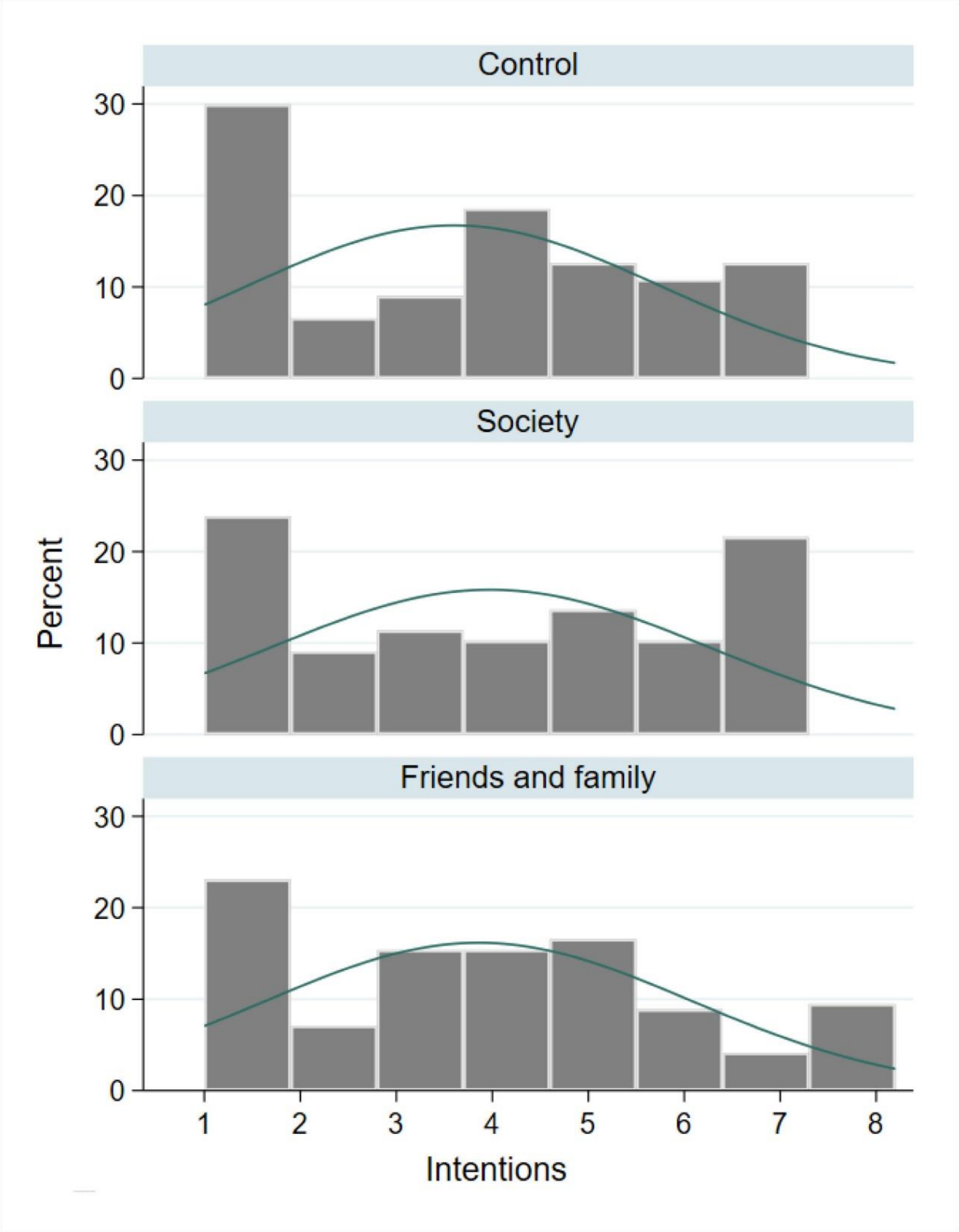

Figure S2. Distribution of perceived social importance of the vaccine across the experimental conditions

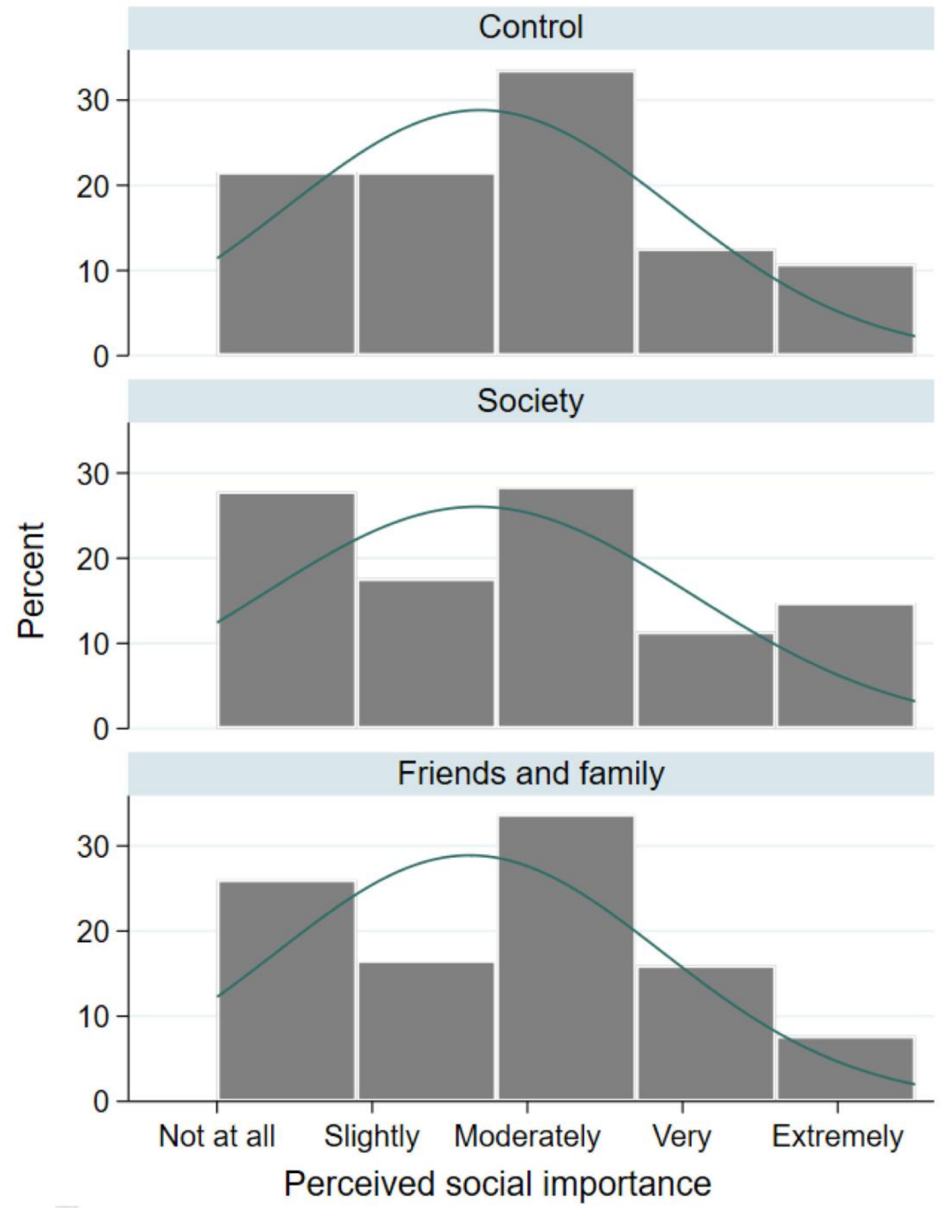

Figure S3. Distribution of the expected vaccination uptake across the experimental conditions

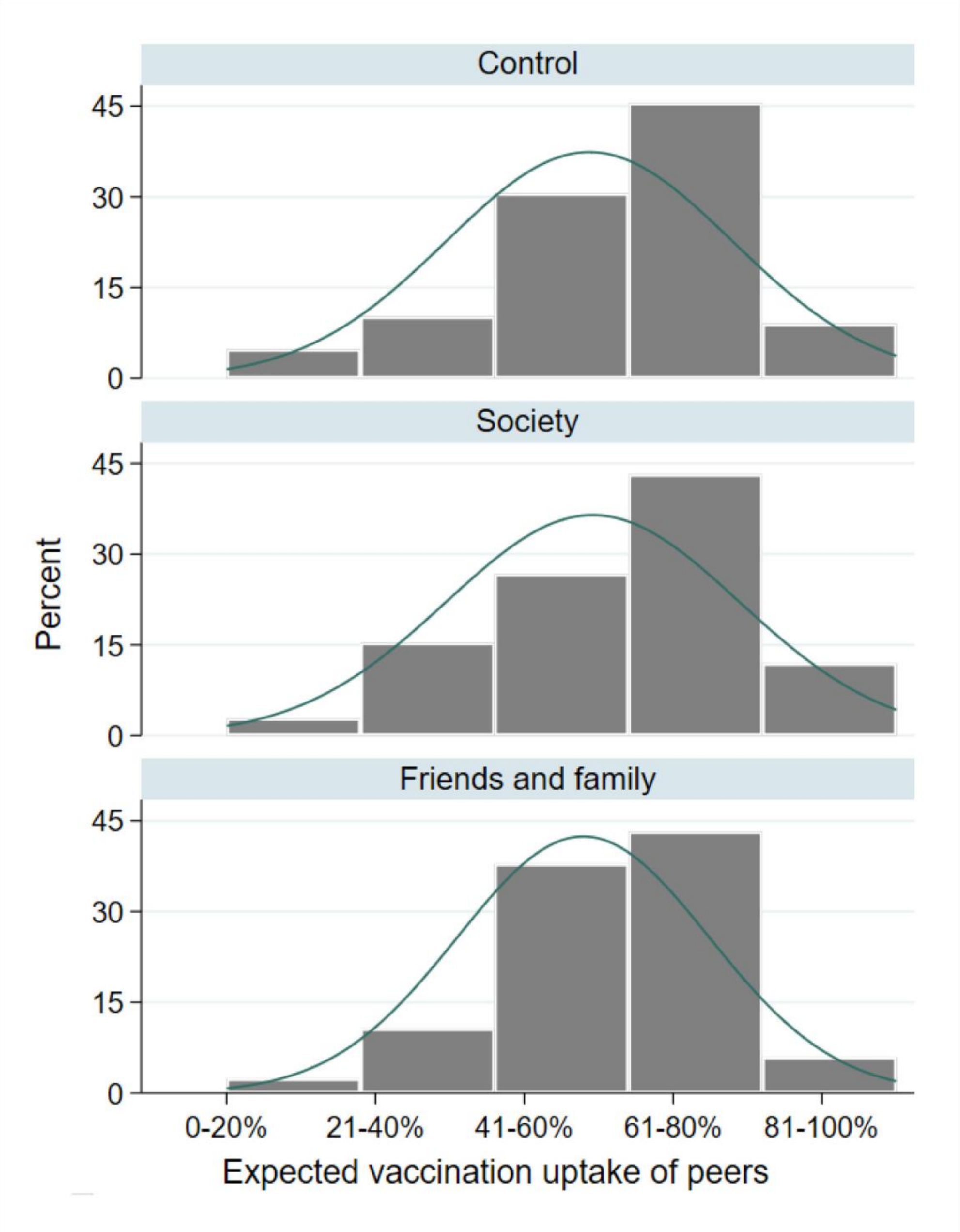

Figure S4. Attitudes towards vaccination

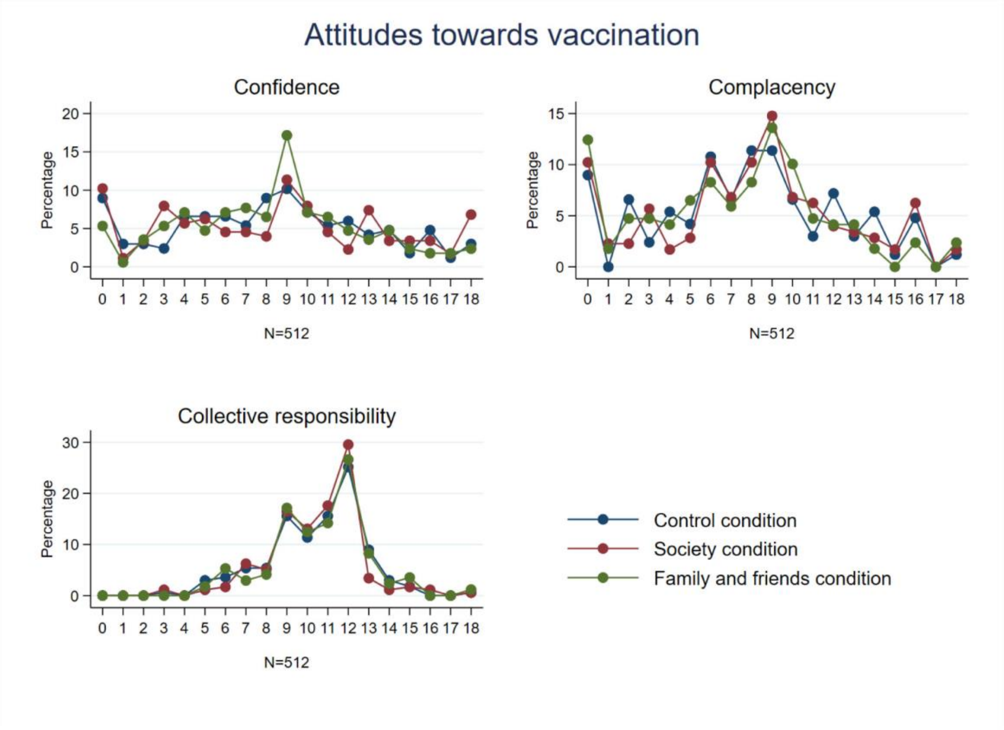

Supplement: Supplementary data [file bmjopen-2022-065804supp003.pdf]
