# Supplementary material for: Effect of communicating community immunity on COVID-19 vaccine-hesitant people from ethnically diverse backgrounds: an experimental vignette study in the UK
Source: BMJ Open. 2022 Nov 3;12(11):e065804. doi: 10.1136/bmjopen-2022-065804 (PMC9638751; doi:10.1136/bmjopen-2022-065804)
Supplement: Author's manuscript [file bmjopen-2022-065804.draft_revisions.pdf]

BMJ Open

BMJ Open is committed to open peer review. As part of this commitment we make the peer review history of every article we publish publicly available.

When an article is published we post the peer reviewers' comments and the authors' responses online. We also post the versions of the paper that were used during peer review. These are the versions that the peer review comments apply to.

The versions of the paper that follow are the versions that were submitted during the peer review process. They are not the versions of record or the final published versions. They should not be cited or distributed as the published version of this manuscript.

BMJ Open is an open access journal and the full, final, typeset and author-corrected version of record of the manuscript is available on our site with no access controls, subscription charges or pay-per-view fees (http://bmjopen.bmj.com).

If you have any questions on BMJ Open's open peer review process please email info.bmjopen@bmj.com

BMJ Open

The effect of communicating community immunity on COVID-19 vaccine hesitant people from ethnically diverse backgrounds: an experimental vignette study in the UK

Journal:	*BMJ Open*
Manuscript ID	bmjopen-2022-065804
Article Type:	Original research
Date Submitted by the Author:	20-Jun-2022
Complete List of Authors:	Stoffel, Sandro T.; UCL, Research Department of Behavioural Science and Health; University of Basel<br>Kaushal, Aradhna; University College London, Behavioural Science and Health<br>Grimani, Aikaterini; University of Warwick, Warwick Business School<br>von Wagner, Christian; University College London Department of Behavioural Science and Health<br>Sniehotta, Falko F.; Newcastle University; Heidelberg University, Department of Public Health, Preventive and Social Medicine<br>Vlaev, Ivo; Warwick Business School
Keywords:	COVID-19, PUBLIC HEALTH, INFECTIOUS DISEASES

SCHOLARONE™
Manuscripts

I, the Submitting Author has the right to grant and does grant on behalf of all authors of the Work (as defined in the below author licence), an exclusive licence and/or a non-exclusive licence for contributions from authors who are: i) UK Crown employees; ii) where BMJ has agreed a CC-BY licence shall apply, and/or iii) in accordance with the terms applicable for US Federal Government officers or employees acting as part of their official duties; on a worldwide, perpetual, irrevocable, royalty-free basis to BMJ Publishing Group Ltd ("BMJ") its licensees and where the relevant Journal is co-owned by BMJ to the co-owners of the Journal, to publish the Work in this journal and any other BMJ products and to exploit all rights, as set out in our licence.

The Submitting Author accepts and understands that any supply made under these terms is made by BMJ to the Submitting Author unless you are acting as an employee on behalf of your employer or a postgraduate student of an affiliated institution which is paying any applicable article publishing charge ("APC") for Open Access articles. Where the Submitting Author wishes to make the Work available on an Open Access basis (and intends to pay the relevant APC), the terms of reuse of such Open Access shall be governed by a Creative Commons licence – details of these licences and which Creative Commons licence will apply to this Work are set out in our licence referred to above.

Other than as permitted in any relevant BMJ Author's Self Archiving Policies, I confirm this Work has not been accepted for publication elsewhere, is not being considered for publication elsewhere and does not duplicate material already published. I confirm all authors consent to publication of this Work and authorise the granting of this licence.

For peer review only

The effect of communicating community immunity on COVID-19 vaccine hesitant people from ethnically diverse backgrounds: an experimental vignette study in the UK

Sandro T. Stoffel[1,2], Aradhna Kaushal[1], Aikaterini Grimani[3], Christian von Wagner[1], Falko F. Sniehotta[4,5], Ivo Vlaev[3]

Correspondence to: Dr Aikaterini Grimani; aikaterini.grimani@wbs.ac.uk

University of Warwick, Warwick Business School, Scarman Rd, CV4 7AL, Coventry, UK

1. Research Department of Behavioural Science and Health, UCL, London, UK
2. Institute for Pharmaceutical Medicine, University of Basel, Basel; Switzerland
3. NIHR Policy Research Unit in Behavioural Science - Behavioural Science Group, Warwick Business School, University of Warwick, Coventry, UK
4. NIHR Policy Research Unit in Behavioural Science - Population Health Sciences Institute, Faculty of Medical Sciences, Newcastle University, Newcastle upon Tyne, UK.
5. Department of Public Health, Preventive and Social Medicine, Center for Preventive Medicine and Digital Health Baden-Wuerttemberg, Heidelberg University, Mannheim, Germany

Words: 2645

Tables: 2

Figures: 1

For peer review only

Abstract

Objectives: Achieving high vaccination coverage is vital to the efforts of curbing the impact the COVID-19 pandemic on public health and society. This study tested whether communicating the social benefit through community protection for friends and family members vs. overall society, affects vaccination intention and perception among a sample enriched with respondents from black and ethnic minority backgrounds.

Design: A web-based experimental survey was conducted. Eligible individuals were individually randomised, with equal probability, to one of three experimental vignettes.

Setting: England

Participants: We recruited 512 (212 white, 300 ethnically diverse) vaccine-hesitant members from an online panel.

Primary and secondary outcome measures: The primary outcome was the intention to get vaccinated against COVID-19. The secondary outcome consisted of a behavioural measure in the form of active interest in reading more about the coronavirus (COVID-19) vaccine. Additional measures included the perceived importance and expected uptake in others, as well as the attitudes towards vaccination.

Results: Logistic regression models did not show an effect of the messages on intentions for the overall sample, communicating the role of vaccination in achieving community immunity yielded higher vaccination intentions among study participants with White ethnic background, but not among respondents from ethnically diverse backgrounds. The messages, however, did not affect perceived importance of the vaccine, expected vaccination uptake and active interest in reading more about the vaccine.

Conclusions: Thus, although highlighting social benefits of COVID-19 vaccinations can increase intentions among vaccine non-intenders, they are unlikely to address barriers among ethnically diverse communities.

For peer review only

Strengths and limitations of this study

- Mentioning the social benefit of vaccination through community protection for friends and family members or overall society only affected vaccination intentions among respondents from a White Ethnic background.
- All ethnic minorities were combined together, thus, nothing can be said about the impact of the messages for different ethnic minorities.
- Vaccination intentions and willingness to read more about the vaccine have been assessed and therefore, the utility of communicating community immunity in changing vaccination behaviour cannot be determined.

For peer review only

Introduction

The SARS-CoV-2 (COVID-19) pandemic has led to over 85 thousand excess deaths in England and Wales over 18 million deaths worldwide [1, 2]. The success of the coronavirus (COVID-19) vaccination program depends on high levels of coverage, around 80%, to achieve community immunity [3]. This is increasingly important as vaccine effectiveness wanes over time and may not be as protective of new COVID-19 variants, requiring booster vaccinations to maintain a high level of coverage [4]. However, some subgroups of the population are more likely to be vaccine hesitant with uptake and intention varying by gender, age, education, employment status, deprivation, and ethnicity [5-7].

Data from the Office for National Statistics show higher rates of vaccine hesitancy in younger age groups, women, people working in hospitality, personal services or transport sectors, people living in more deprived areas, and people from ethnic minority groups, particularly Black/Black British and Pakistani/Bangladeshi ethnic groups [6, 8, 9]. This is particularly concerning as many of these groups are at increased risk of contracting COVID-19, hospitalisation and dying from COVID-19 [10-12]. Reasons for vaccine hesitancy include worry about the side effects, worry about the long-term effects on health, waiting to see how effective vaccines are, and concerns about safety [13].

A key method to promote vaccine uptake among individuals is to design public health messages to promote collective goals and use targeted messages as a mechanism to achieve the same wider collective aim, e.g., save the NHS, protect lives, promote awareness of symptoms and control measures [14-18]. These ‘Protect each other’ messages highlight the benefits of protective behaviours for the group and its most vulnerable members by promoting care for others rather than individual self-interest

For peer review only

[19]. In addition, enlisting trusted voices has been shown to make public health messages more effective in changing behaviour during epidemics [20, 21].

While experimental studies on social preferences have shown that individuals are motivated by the wellbeing of others, individuals may hold social identities at various levels of abstraction, ranging from concrete groups of individuals (e.g., own friends and family) to broader categories of individuals such as citizens of their country [22, 23]. Studies on social preferences have shown that individuals exhibit more prosocial behaviour if they know more about the potential beneficiaries, such as their social belonging [23-26]. Thus, prosocial behaviour is negatively related to social distance; e.g. whether beneficiaries are close friends or not [27-29]. As such, individuals' intentions to get immunized for the benefit of others may depend on the social distance to the beneficiaries of community immunity. A recent experimental study found that people in England had higher intentions to get vaccinated against a hypothetical flu if they were told about the social benefit of vaccination for their close social environment, such as friends and family. Communicating the social benefit for the overall society, however, did not increase vaccination intentions [30]. Furthermore, as the cultural background can influence the perception of social benefits, communicating the concept of herd immunity can increase vaccination intentions among cultures that focus on collective benefits. Prosocial nudges can thus help to close these immunity gaps (22).

The primary aim of the current research was to explore how the framing of community immunity influences vaccination intentions among White and ethnic minorities. For this we conducted a web-based experiment and recruited vaccine hesitant individuals with a White ethnicity an ethnic minority background. Specifically, we investigated whether the definition of the beneficiaries of community immunity affects vaccination intentions, expecting that highlighting the indirect effect of immunization for the close social

For peer review only

environment would increase intention to get a coronavirus (COVID-19) vaccine. In line with previous research, we expect that the information about community immunity to be more effective in increasing vaccination intentions, if it mentions close social environments instead of overall society, as the definition of the beneficiaries influences social preferences [30, 31]. Additionally, the study also investigated whether communicating community immunity influences the perception of the vaccine in terms of social importance and expected uptake of peers.

Methods

General procedure. A web-based experimental survey was programmed in Qualtrics to assess how the framing of community immunity influences vaccination intentions. The survey was conducted in May 2021 and featured a sample of adult men and women who were invited by a survey vendor (Dynata) to take part in an online survey on COVID-19 vaccination. Only study participants who completed the survey received a small financial incentive from the survey vendor, which was defined by the length of the questionnaire.

Survey design. At the start of the survey, study participants were asked to give explicit consent for their data to be used and published as part of this research project before they could continue in the survey. A first screener question on screening behaviour was used to recruit individuals who had either been invited for the vaccination but declined it or who have not been invited yet but did not intend to get vaccinated when invited. The question was adapted from the NIHR Policy Research Unit Behavioural Sciences questionnaire on vaccination intentions. Participants were told that they would have the vaccine once it is available to them, with the response options; strongly disagree, disagree, neither agree nor disagree, agree and strongly agree [32]. Individuals who disagreed or neither agreed nor disagreed were then asked about their

For peer review only

ethnicity. As vaccine hesitancy was identified as being common among ethnic minority groups, a quota was employed on White ethnicity to recruit no more than 215 respondents from a White ethnic background and at least 300 study participants with an ethnic minority background so that meaningful differences could be explored. Individuals who were filtered out from the survey were redirected to the study briefing and final survey page where they were thanked for their participation (see Figure 1).

< Figure 1 here >

Experimental conditions. Eligible individuals were individually randomised, with equal probability, to one of three experimental vignettes with different versions of the COVID-19 vaccination invitation letter. Each participant was told that the vaccination would be free and consist of two separate shots that would be administered in a vaccination centre. The type of vaccine was not mentioned in the vignettes. The last sentence of the vignette featured the experimental manipulation with the description of the beneficiaries of the vaccination (see Table 1).

Table 1. Behavioural messages tested in the experiment

**Condition**	**Final paragraph of the vignette**
Control	Get vaccinated to protect you from Covid-19.
Society	Get vaccinated to protect you and the vulnerable people in Great Britain from Covid-19.
Family and friends	Get vaccinated to protect you, your community, and your vulnerable friends and family members such as your children, your parents or your grandparents from the coronavirus (COVID-19).

Ethics approval and preregistration. The research project received ethical approval of the University of Warwick's Humanities and Social Sciences Research Ethics Committee (HSSREC 142/20-21). The design and analysis plan for the experiment

For peer review only

were preregistered on Open Science Framework (OSF; https://osf.io/k4jyv/?view_only=08694ea40f994671b2dd899ae656f34e) prior to any data being collected. Analysis scripts and an abridged dataset containing all primary study variables can also be found on OSF.

Outcome measures

Vaccination intentions. The primary outcome was the intention to get vaccinated against COVID-19. This was measured using the question: "Would you get vaccinated against the coronavirus?" with responses on a partially labelled eight-point scale, where 1 means that one will definitely not get vaccinated and 8 indicates that one will definitely get vaccinated [30]. The response options of these questions were taken from previous studies [33, 34]. Individuals who stated low vaccination intentions (below 5) were asked to indicate reasons for not wanting to get vaccinated (see Supplementary Table S1 for answers).

Active interest. The secondary outcome consisted of a behavioural measure in the form of active interest in reading more about the coronavirus (COVID-19) vaccine [35, 36]. Following the experimental manipulation towards the end of the survey, participants were asked whether they would like to 'read' or 'skip' information about the vaccination procedure before completing the survey. Those who indicated that they would like to skip it were sent to the end of the questionnaire with the socio-demographic question, while those who wanted to read the information were presented additional information about the vaccination from the official NHS website. Choosing to read the information was interpreted as active interest in reading more health information. Engagement with the information was measured with four comprehension questions.

For peer review only

Perceived importance and expected uptake in others. To explore the impact of the experimental manipulation on the perceived importance of the immunization programme, we included the question. “Do you believe that getting vaccinated against the Covid-19 is socially important?”. Additionally, we asked whether they thought that most people would get immunized with the question “Do you think that most people will get vaccinated?” Both items featured five-point fully-labelled Likert scale response options [30].

Attitudes towards vaccination. Nine additional questions from the 5C measure of psychological antecedents of vaccination were included as further outcomes [37]. The questions featured 7-point fully-labelled Likert-scales [0;6] and were combined into three constructs on confidence in vaccination and public authorities, complacency and perceived collective responsibility with scores between 0 and 18 each.

Sample size calculation and statistical analysis

The sample size for the experiment was based on estimates from a previous study [30]. A sample size calculation suggested a minimum group sizes of 170 participants per group (510 participants in total) [30, 38]. We used multivariable ordered and binary logistic regressions adjusting for baseline vaccination invitation status and sociodemographic variables to investigate the effect of the experimental conditions on vaccination intentions and active interest. The regressions were run on the whole sample and on the two ethnic subgroups; White and BAME (Black, Asian and minority ethnic) which describes non-white ethnicities. The statistical analysis was conducted with Stata/IC version 16.0 (StataCorp LP, College Station, TX).

Patient and public involvement (PPI)

No patient or public were involved in the design, conduct and reporting of this research.

For peer review only

Results

Study sample. The analytical sample comprised 512 individuals who had not yet been invited for a COVID-19 vaccination appointment but were planning not to accept the invitation (N=302; 59.0%) or have been invited and had declined the vaccination (N=210, 41.0%) More than half of the study participants were from an ethnic minority (excluding white minorities) group (N=307, 59.6%), female (N=285, 55.7%) and between 25 and 34 years old (N=236, 46.1%). There were no statistically significant differences in sociodemographic characteristics, indicating that there were no imbalances due to levels of drop-out varying among the three experimental conditions (see Table S2 in the supplementary file for the characteristics of the study sample).

Vaccination intentions. The results of the ordered logistic regression, reported in Table 2, showed that, compared with the control condition, neither the society nor the family and friends message yielded higher vaccination intentions (see Figure S1 in supplementary file for the distribution). Looking at White and ethnic minority subgroups separately reveals that the behavioural messages were associated with higher vaccination intentions in the former but not the latter group (adjusted odds ratio (aOR): 1.94; 95% confidence interval (CI): 1.07–3.51 and aOR: 2.07; 95% CI: 1.08–3.96).

For peer review only

Table 2: Ordered logistic regression on vaccination intentions [1;8]

	Mean	Model 1: Overall sample OR	95% CI	aOR	95% CI	Mean	Model 2: Only White ethnic background OR	95% CI	aOR	95% CI	Mean	Model 3: Black, Asian, mixed or other ethnic background OR	95% CI	aOR	95% CI
Condition															
Control	3.60	Ref.		Ref.		3.42	Ref.		Ref.		3.74	Ref.		Ref.	
Society	3.98	1.362	0.936 - 1.981	1.284	0.876 - 1.880	4.27	1.965	1.103 - 3.498*	1.937	1.069 - 3.512*	3.74	0.999	0.609 - 1.638	0.952	0.577 - 1.571
Family and friends	3.86	1.200	0.826 - 1.744	1.317	0.893 - 1.942	3.89	1.430	0.779 - 2.627	2.066	1.077 - 3.962*	3.84	1.030	0.638 - 1.663	1.056	0.644 - 1.732
Vaccination status															
Invited	3.70	Ref.		Ref.		3.85	Ref.		Ref.		3.59	Ref.		Ref.	
Not invited	3.98	1.263	0.929 - 1.718	1.192	0.859 - 1.654	3.90	1.035	0.636 - 1.683	0.678	0.394 - 1.169	4.02	1.450	0.974 - 2.161	1.516	0.993 - 2.315
Age															
18-24 years old	3.97	Ref.		Ref.		4.42	Ref.		Ref.		3.75	Ref.		Ref.	
25-34 years old	4.04	1.075	0.753 - 1.536	0.932	0.632 - 1.375	4.15	0.839	0.465 - 1.512	0.694	0.370 - 1.304	3.94	1.179	0.743 - 1.872	0.968	0.584 - 1.604
35-44 years old	3.84	0.910	0.549 - 1.509	0.743	0.426 - 1.296	2.93	0.346	0.120 - 0.996*	0.289	0.089 - 0.945*	4.08	1.311	0.726 - 2.366	1.085	0.567 - 2.076
45+ years old	2.58	0.297	0.173 - 0.508**	0.289	0.161 - 0.519**	2.24	0.182	0.077 - 0.430**	0.163	0.064 - 0.416**	2.86	0.423	0.209 - 0.855*	0.347	0.159 - 0.755**
Gender															
Male	4.08	Ref.		Ref.		4.25	Ref.		Ref.		3.93	Ref.		Ref.	
Female	3.62	0.697	0.511 - 0.953*	0.653	0.473 - 0.901**	3.50	0.562	0.344 - 0.917*	0.528	0.319 - 0.876*	3.68	0.807	0.536 - 1.215	0.768	0.503 - 1.174
Non-binary	3.00	0.480	0.120 - 1.927	0.358	0.082 - 1.553	3.00	0.422	0.063 - 2.802	0.394	0.045 - 3.447	3.00	0.512	0.069 - 3.820	0.401	0.050 - 3.185
Ethnicity															
White	3.87	Ref.		Ref.					.			.		.	
Mixed	3.46	0.741	0.461 - 1.192	0.687	0.419 - 1.126										
Asian	4.42	1.509	0.998 - 2.281	1.477	0.959 - 2.277										
Black	3.43	0.713	0.479 - 1.061	0.673	0.445 - 1.018										
Arab or other	3.77	0.904	0.324 - 2.525	1.017	0.338 - 3.059										
A-levels															
No	3.33	Ref.		Ref.		3.13	Ref.		Ref.		3.52	Ref.		Ref.	
Yes	3.94	1.664	1.132 - 2.444**	1.609	1.064 - 2.431*	4.12	2.033	1.167 - 3.542*	2.095	1.143 - 3.838*	3.83	1.323	0.769 - 2.275	1.285	0.724 - 2.283
Living condition															
Alone	3.72	Ref.		Ref.		4.00	Ref.		Ref.		3.57	Ref.		Ref.	
With somebody	3.92	1.170	0.861 - 1.591	1.207	0.861 - 1.693	3.76	0.840	0.519 - 1.361	0.806	0.485 - 1.341	4.07	1.533	1.025 - 2.293*	1.815	1.143 - 2.884*
Paid employment															
No	3.52	Ref.		Ref.		3.43	Ref.		Ref.		3.57	Ref.		Ref.	
Yes	3.94	1.366	0.984 - 1.895	1.174	0.828 - 1.664	4.03	1.521	0.896 - 2.580	1.229	0.700 - 2.157	3.86	1.273	0.837 - 1.935	1.064	0.678 - 1.671
N		512		512			207		207			305		305	

* *p*<0.05; ** *p*<0.01

For peer review only

Active interest. The experimental manipulations did not influence interest in reading more about the vaccination process (see Supplementary Table S3 for the regression results). Independently from the experimental condition, around one third of the respondents stated that they wanted to read more (34.7–44.9%, $\chi 2(2, N=512)=3.991, p=0.136$). A Kruskal-Wallis test did not reveal any differences in vaccination knowledge across the conditions ($\chi 2=0.655$, $p=0.721, df=2$). Most participants (45.9%) who read the additional information answered 2 out of 4 comprehension questions correctly.

Perceived importance and expected uptake in others. Only a minority of participants stated that they perceived the vaccinations as not important (N=129, 25.2%) and most thought the vaccination to be moderately important (N=163, 31.8%). Across the three conditions, the majority (N=271, 52.9%) expected that more than 60% of their peers would get vaccinated. Importantly, the experimental manipulations did not affect the perceived importance of the vaccination nor the expected uptake (see Supplementary Table S4, S5 and S6 for the regression results and Figures S2 and S3 for the distributions).

Attitude towards vaccination. The median values for confidence in vaccination and public authorities was only 9 out of 18 (SD 4.84) and did not vary between experimental conditions ($\chi^2 = 0.05$, $p = 0.974$, $df = 2$). Similarly, complacency, the feeling that vaccination is not necessary was also low relatively with a median of 8 out of 18 (SD 4.51) and also no variation between experimental conditions ($\chi^2 = 2.48$, $p = 0.290$, $df = 2$). The median for perceived social responsibility was 11 out of 18 (SD 2.30) and was also not influenced by the experimental manipulations ($\chi^2 = 0.78$, $p = 0.679$, $df = 2$). See Figure S4 in the supplementary file for the distribution of the attitude scores.

For peer review only

Reasons for vaccination hesitancy. Among the 306 study participants who did not intend to be vaccinated, fear of side effect and doubts about the protective nature of the vaccine were the most frequently mentioned reasons to not get vaccinated (66%) followed by not believing that the vaccine offers protection (38%). See Table S1 in the supplementary file for a full list of the stated reasons.

General discussion

This is one of the first studies to investigate how the framing of the beneficiaries of community immunity influences vaccination intentions [30, 31]. In a web-based experimental study, we tested whether mentioning the social benefit of vaccination through community protection for friends and family members or overall society affects COVID-19 vaccination behaviour among white and ethnic minorities. Contrary to a previous study (Stoffel and Herrmann, 2021), adding this information only affected vaccination intentions among respondents from a White Ethnic background. This lack of effect among respondents with from ethnic minorities may be due to previously reported high levels of medical mistrust in ethnic minority groups which was found to mediate participation of Black participants in COVID-19 vaccine trials and uptake more generally [39]. Future research could explore this further by testing messages relating to vaccine safety and efficacy.

Limitations of the experimental study

Our study has several limitations which call for follow-up research. Firstly, we only had a small analytical sample due to the difficulties in recruiting vaccine-hesitant study participants in the ongoing vaccination programme, limiting statistical power. Secondly, we had to combine all ethnic minorities together so that nothing can be said about the impact of the messages for different ethnic minorities. Thirdly, we assessed vaccination intentions and willingness to read more about the vaccine. Therefore, the

For peer review only

utility of communicating community immunity in changing vaccination behaviour cannot be determined as intentions do not necessarily translate to behaviours [40, 41]. Thus, additional strategies may be required to build on motivational changes to increase vaccination uptake, such as implementation intentions [42].

Conclusion

This study tested how the framing of community immunity affects intentions to get the COVID-19 vaccine among vaccine-hesitant individuals. Focusing on ethnic minority groups, the study investigated whether behavioural messages appealing to get vaccinated to protect oneself, family members and friends or the overall society increased vaccination intentions and interest in reading more about the vaccine. As the behavioural messages failed to affect vaccination behaviour of vaccine-hesitant participants, future studies should develop interventions that are specifically targeted at reducing fear about side effects and concerns about the protective nature of the COVID-19 vaccine in order to achieve the highest possible level of vaccination uptake.

For peer review only

Contributorship statement

Conceptualisation: SS, CVW, AK, AG, FFS, IV; Data curation: AK, SS; Formal analysis, AK, SS, IV; Investigation: SS, AG, CVW, AK, FFS, IV; Methodology: SS, AG, CVW, AK, FFS, IV; Supervision, FFS, IV; Writing – original draft: SS, AK; Writing – review & editing: SS, CVW, AK, AG, FFS, IV.

Competing Interests statement

The authors declare no conflict of interest.

Funding Statement

This project is funded by the National Institute for Health Research (NIHR) [Policy Research Program (Policy Research Unit in Behavioural Science PR-PRU1217-20501)]. The views expressed are those of the author(s) and not necessarily those of the NIHR or the Department of Health and Social Care.

Data availability statement

The data presented in this study are available upon reasonable request from the corresponding author.

For peer review only

References

1. Islam, N., et al., *Excess deaths associated with covid-19 pandemic in 2020: age and sex disaggregated time series analysis in 29 high income countries.* bmj, 2021. **373**.

2. Wise, J., *Covid-19: Global death toll may be three times higher than official records, study suggests*. 2022, British Medical Journal Publishing Group.

3. Mills, M., et al., *COVID-19 vaccine deployment: Behaviour, ethics, misinformation and policy strategies.* London: The Royal Society & The British Academy, 2020.

4. Feikin, D.R., et al., *Duration of effectiveness of vaccines against SARS-CoV-2 infection and COVID-19 disease: results of a systematic review and meta-regression.* The Lancet, 2022.

5. Martin, C.A., et al., *Association of demographic and occupational factors with SARS-CoV-2 vaccine uptake in a multi-ethnic UK healthcare workforce: a rapid real-world analysis.* MedRxiv, 2021.

6. Robertson, E., et al., *Predictors of COVID-19 vaccine hesitancy in the UK household longitudinal study.* Brain, behavior, and immunity, 2021. **94**: p. 41-50.

7. Neumann-Böhme, S., et al., *Once we have it, will we use it? A European survey on willingness to be vaccinated against COVID-19*. 2020, Springer. p. 977-982.

8. Lockyer, B., et al., *Understanding COVID-19 misinformation and vaccine hesitancy in context: Findings from a qualitative study involving citizens in Bradford, UK.* Health Expectations, 2021. **24**(4): p. 1158-1167.

For peer review only

9. Public Health England, *Disparities in the risk and outcomes of COVID-19*. 2020, Public Health England London.

10. Office for National Statistics. *Why have Black and South Asian people been hit hardest by COVID-19?* . 2020 8th February 2021]; Available from: https://www.ons.gov.uk/peoplepopulationandcommunity/healthandsocialcare/conditionsanddiseases/articles/whyhaveblackandsouthasianpeoplebeenhithardestbycovid19/2020-12-14

11. Aldridge, R.W., et al., *Black, Asian and Minority Ethnic groups in England are at increased risk of death from COVID-19: indirect standardisation of NHS mortality data.* Wellcome open research, 2020. **5**.

12. Paul, E., A. Steptoe, and D. Fancourt, *The Lancet Regional Health—Europe Attitudes towards vaccines and intention to vaccinate against COVID-19: Implications for public health communications.* Lancet Reg. Health Eur, 2021. **1**.

13. Office for National Statistics. *Coronavirus and vaccine hesitancy, Great Britain: 13 January to 7 February 2021*. 2021 11th November 2021]; Available from: https://www.ons.gov.uk/peoplepopulationandcommunity/healthandsocialcare/healthandwellbeing/bulletins/coronavirusandvaccinehesitancygreatbritain/13januaryto7february2021.

14. Kelly, B.J. and R.C. Hornik, *Effects of framing health messages in terms of benefits to loved ones or others: An experimental study.* Health Communication, 2016. **31**(10): p. 1284-1290.

For peer review only

15. Li, M., et al., *Stimulating influenza vaccination via prosocial motives.* PloS one, 2016. **11**(7): p. e0159780.

16. Ghio, D., et al., *What influences people's responses to public health messages for managing risks and preventing infectious diseases? A rapid systematic review of the evidence and recommendations.* BMJ open, 2021. **11**(11): p. e048750.

17. Jordan, J.J., E. Yoeli, and D.G. Rand, *Don't get it or don't spread it: Comparing self-interested versus prosocial motivations for COVID-19 prevention behaviors.* Scientific reports, 2021. **11**(1): p. 1-17.

18. Everett, J.A., et al., *The effectiveness of moral messages on public health behavioral intentions during the COVID-19 pandemic.* 2020.

19. Moran, M.B. and S. Sussman, *Translating the link between social identity and health behavior into effective health communication strategies: An experimental application using antismoking advertisements.* Health communication, 2014. **29**(10): p. 1057-1066.

20. Abu-Akel, A., A. Spitz, and R. West, *The Effect of Spokesperson Attribution on Public Health Messaging During the COVID-19 Pandemic.* 2020.

21. Vaughan, E. and T. Tinker, *Effective health risk communication about pandemic influenza for vulnerable populations.* American journal of public health, 2009. **99**(S2): p. S324-S332.

22. Andreoni, J., W. Harbaugh, and L. Vesterlund, *Altruism in experiments. The new palgrave dictionary in economics*. 2008, Macmillan, New York.

For peer review only

23. Charness, G. and U. Gneezy, *What's in a name? Anonymity and social distance in dictator and ultimatum games.* Journal of Economic Behavior & Organization, 2008. **68**(1): p. 29-35.

24. Eckel, C.C. and P.J. Grossman, *Altruism in anonymous dictator games.* Games and economic behavior, 1996. **16**(2): p. 181-191.

25. Bohnet, I. and B.S. Frey, *Social distance and other-regarding behavior in dictator games: Comment.* American Economic Review, 1999. **89**(1): p. 335-339.

26. Burnham, T.C., *Engineering altruism: A theoretical and experimental investigation of anonymity and gift giving.* Journal of Economic Behavior & Organization, 2003. **50**(1): p. 133-144.

27. Jones, B. and H. Rachlin, *Social discounting.* Psychological science, 2006. **17**(4): p. 283-286.

28. Leider, S., et al., *Directed altruism and enforced reciprocity in social networks.* The Quarterly Journal of Economics, 2009. **124**(4): p. 1815-1851.

29. Goeree, J.K., A. Riedl, and A. Ule, *In search of stars: Network formation among heterogeneous agents.* Games and Economic Behavior, 2009. **67**(2): p. 445-466.

30. Stoffel, S. and B. Herrmann, *How Does Communicating Herd Immunity Affect Immunization Intentions?: Evidence From a Cross-Country Online Experiment.* International Journal of Applied Behavioral Economics (IJABE), 2021. **10**(4): p. 12-20.

For peer review only

31. Betsch, C., et al., *On the benefits of explaining herd immunity in vaccine advocacy.* Nature human behaviour, 2017. **1**(3): p. 1-6.

32. Kelly, M., et al., *Factors associated with vaccine intention in adults living in England who either did not want or had not yet decided to be vaccinated against COVID-19.* 2021.

33. Klett-Tammen, C.J., et al., *Determinants of tetanus, pneumococcal and influenza vaccination in the elderly: a representative cross-sectional study on knowledge, attitude and practice (KAP).* BMC public health, 2015. **16**(1): p. 1-9.

34. Loubet, P., et al., *Pneumococcal and influenza vaccine uptake in adults with inflammatory bowel disease in France: Results from a web-based study.* Digestive and Liver Disease, 2018. **50**(6): p. 563-567.

35. Stoffel, S.T., et al., *Testing the decoy effect to increase interest in colorectal cancer screening.* PloS one, 2019. **14**(3): p. e0213668.

36. Stoffel, S.T., et al., *Testing verbal quantifiers for social norms messages in cancer screening: evidence from an online experiment.* BMC public health, 2019. **19**(1): p. 1-11.

37. Betsch, C., et al., *Beyond confidence: Development of a measure assessing the 5C psychological antecedents of vaccination.* PloS one, 2018. **13**(12): p. e0208601.

38. Cohen, J., *Stafisfical power analysis for the behavioural sciences (2nd edn.). Hillside*. 1988, NJ: Erlbaum.

For peer review only

39. Thompson, H.S., et al., *Factors associated with racial/ethnic group–based medical mistrust and perspectives on COVID-19 vaccine trial participation and vaccine uptake in the US.* JAMA Network Open, 2021. **4**(5): p. e2111629-e2111629.

40. Sheeran, P., *Intention—behavior relations: a conceptual and empirical review.* European review of social psychology, 2002. **12**(1): p. 1-36.

41. Webb, T.L. and P. Sheeran, *Does changing behavioral intentions engender behavior change? A meta-analysis of the experimental evidence.* Psychological bulletin, 2006. **132**(2): p. 249.

42. Milkman, K.L., et al., *Using implementation intentions prompts to enhance influenza vaccination rates.* Proceedings of the National Academy of Sciences, 2011. **108**(26): p. 10415-10420.

For peer review only

Figure 1. Flow through experiment

For peer review only

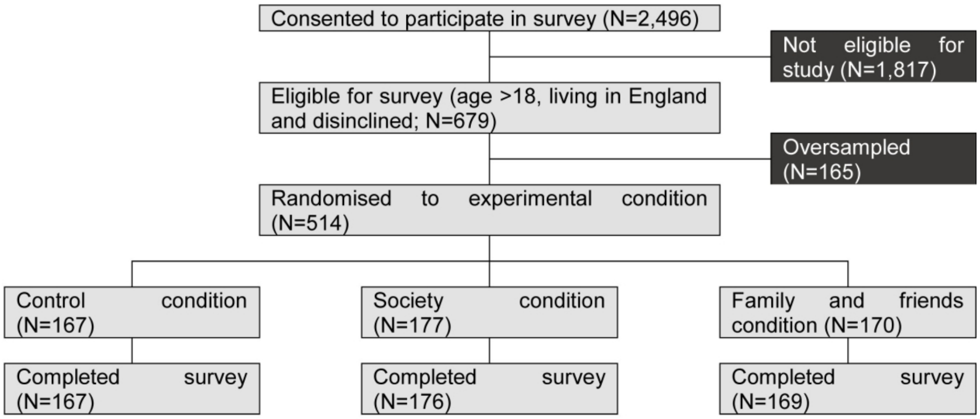

Flow through experiment

186x99mm (300 x 300 DPI)

Supplementary figures

Figure S1. Distribution of vaccination intentions across the experimental conditions

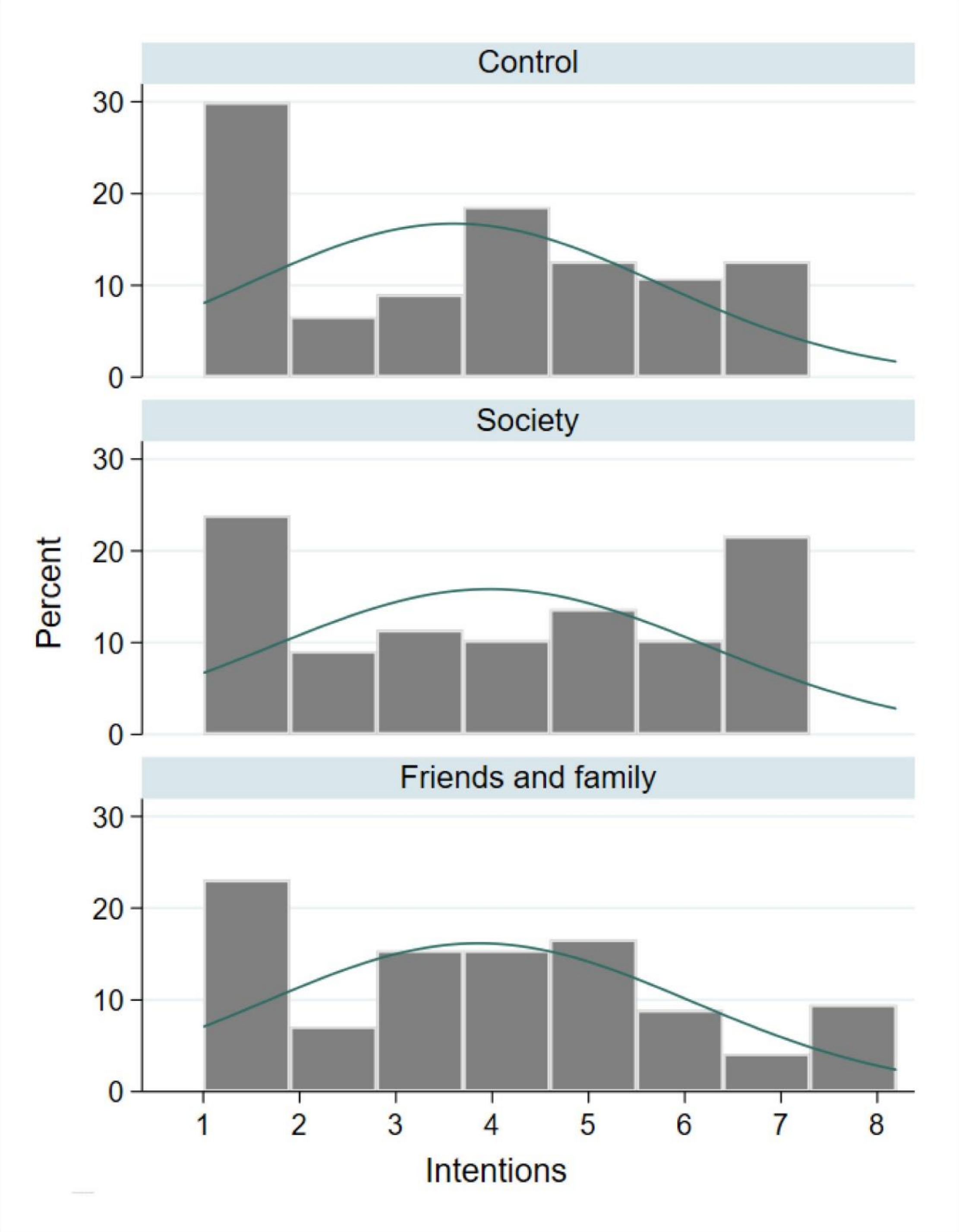

Figure S2. Distribution of perceived social importance of the vaccine across the experimental conditions

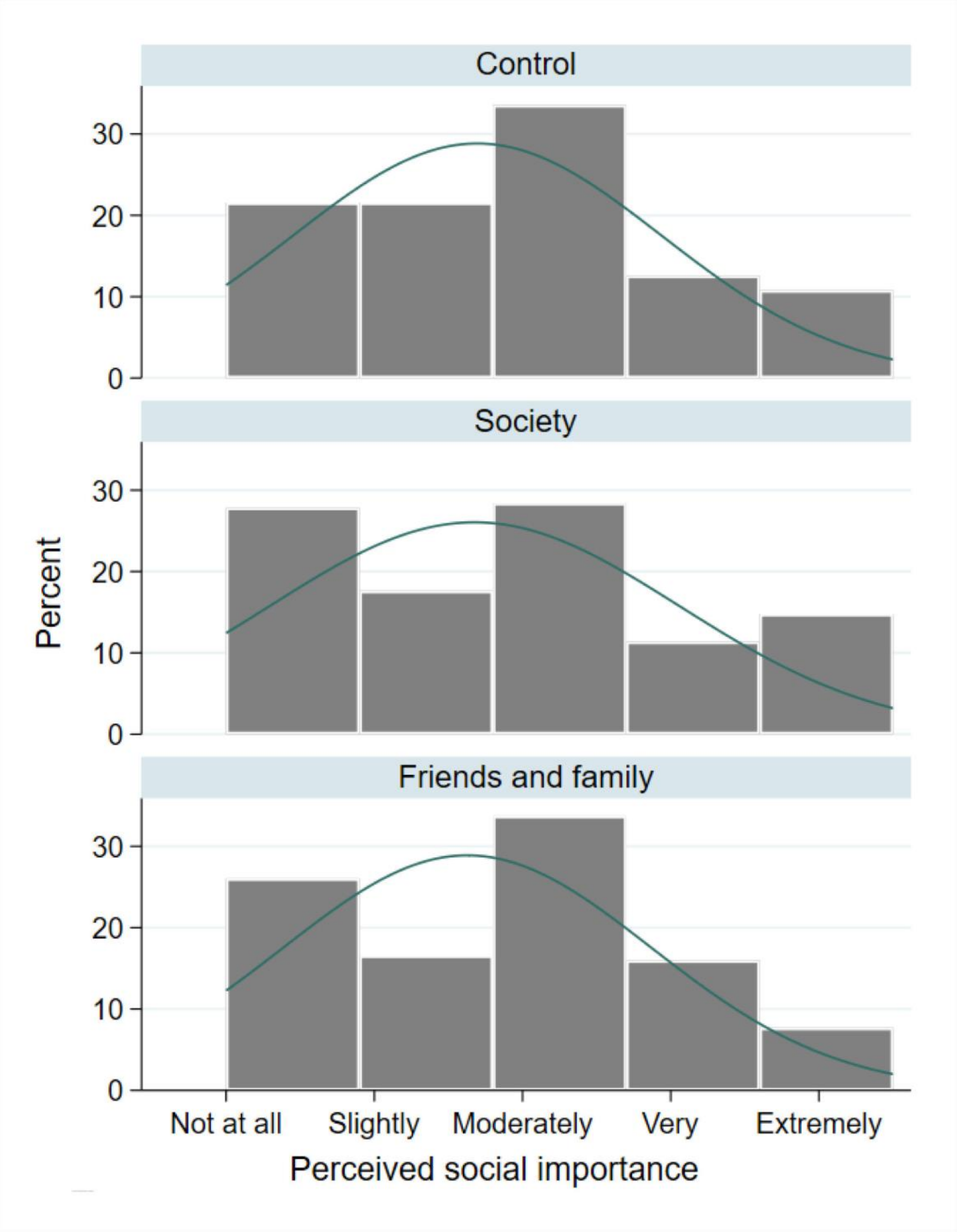

Figure S3. Distribution of the expected vaccination uptake across the experimental conditions

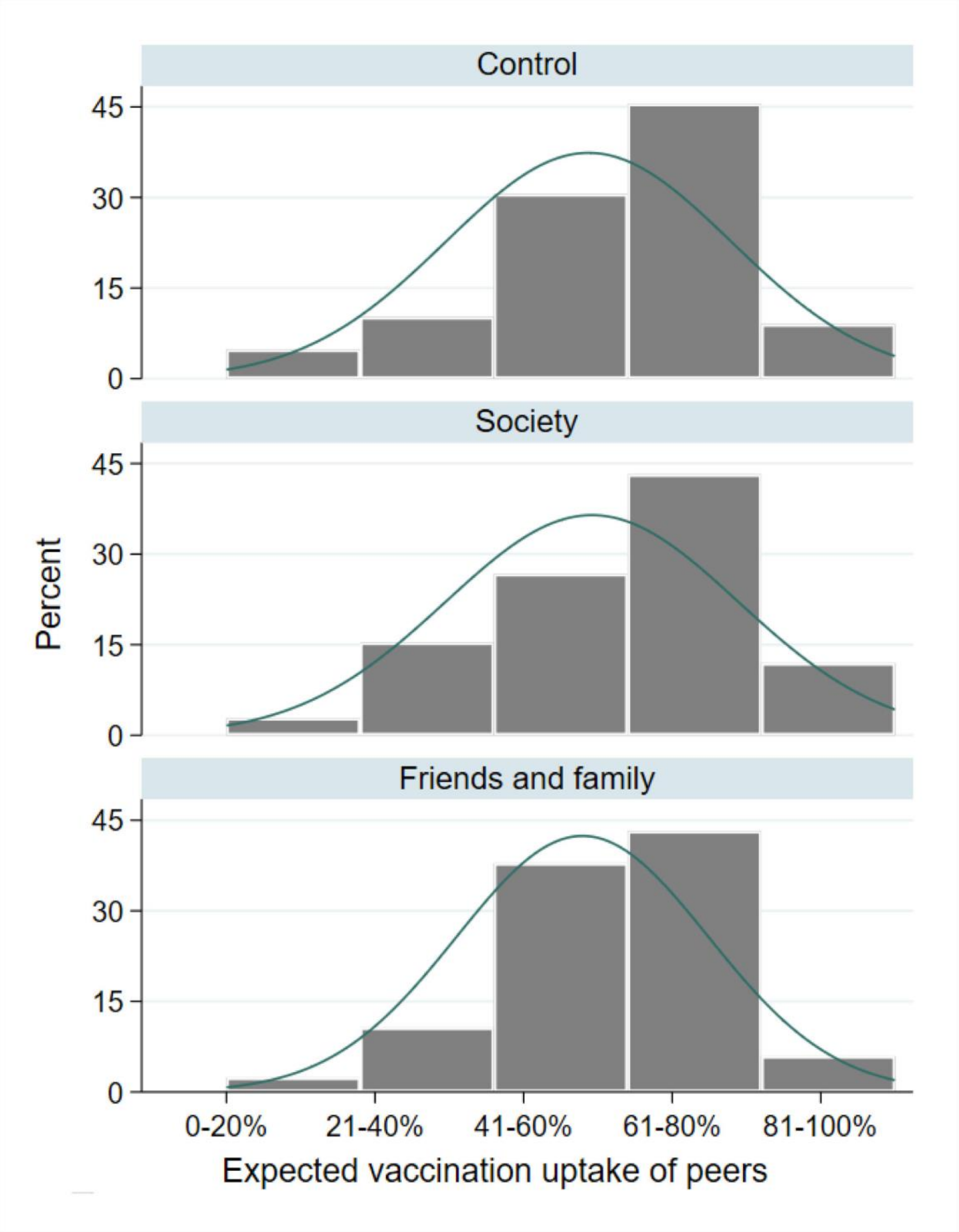

Figure S4. Attitudes towards vaccination

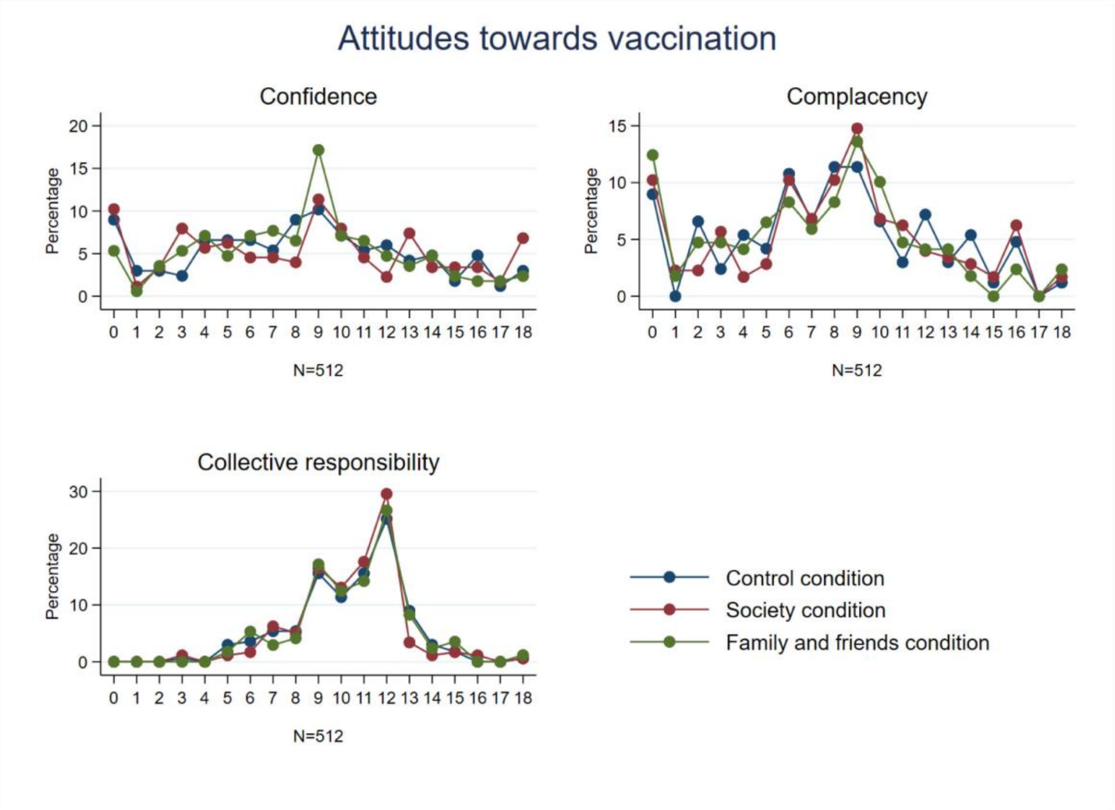

Supplementary tables

Table S1: Reasons for not wanting to get vaccinated after experimental manipulation*

Reason	Overall sample (N=306) N	(%)	Only White ethnic background (N=118) N	(%)	Model 3: Black, Asian, mixed or other ethnic background (N=188) N	(%)
Fear of side effect	201	65.9	83	70.9	118	62.8
Vaccine does not offer protection	115	37.7	47	40.2	68	36.2
Critical reports in media	69	22.6	25	21.4	44	23.4
General objections against vaccination	63	20.7	28	23.9	35	18.6
Fear of needles	44	14.4	20	17.1	24	12.8
Other reasons	37	12.1	16	13.7	21	11.2
Disease is harmless	30	9.8	8	6.8	22	11.7
Discouraged by family/friends' opinion	26	8.5	9	7.7	17	9.0
Discouraged by physician's opinion	17	5.6	9	7.7	8	4.3
Difficulties getting to the appointment	14	4.6	6	5.1	8	4.3
Too much effort	8	2.6	2	1.7	6	3.2

*only answered by study participants that stated low vaccination intentions [1-4] after the experimental manipulation

For peer review only

Table S2: Descriptive statistics across experimental conditions

	Control (N=167)		Society (N=176)		Family and friends (N=169)		Total (N=512)	
	N	(%)	N	(%)	N	(%)	N	(%)
Vaccination status								
Have been invited	101	(60.5%)	94	(53.4%)	107	(63.3%)	302	(59.0%)
Not been invited yet	66	(39.5%)	82	(46.6%)	62	(36.7%)	210	(41.0%)
Age								
18-24 years old	54	(32.3%)	52	(29.5%)	43	(25.4%)	149	(29.1%)
25-34 years old	74	(44.3%)	90	(51.1%)	72	(42.6%)	236	(46.1%)
35-44 years old	14	(8.4%)	14	(8.0%)	35	(20.7%)	63	(12.3%)
45+ years old	25	(15.0%)	20	(11.3%)	19	(11.2%)	64	(12.5%)
Gender								
Male	74	(44.3%)	81	(46.0%)	67	(39.6%)	222	(43.4%)
Female	93	(55.7%)	93	(52.8%)	99	(58.6%)	285	(55.7%)
Non-binary	0	(0.0%)	2	(1.1%)	3	(1.8%)	5	(1.0%)
Ethnicity								
White	72	(43.1%)	78	(44.3%)	57	(33.7%)	207	(40.4%)
Mixed	16	(9.6%)	30	(17.0%)	23	(13.6%)	69	(13.5%)
Asian	30	(18.0%)	34	(19.3%)	36	(21.3%)	100	(19.5%)
Black	45	(26.9%)	32	(18.2%)	46	(27.2%)	123	(24.0%)
Arab or other	4	(2.4%)	2	(1.1%)	7	(4.1%)	13	(2.5%)
A-levels								
No	29	(17.4%)	31	(17.6%)	46	(27.2%)	106	(20.7%)
Yes	138	(82.6%)	145	(82.4%)	123	(72.8%)	406	(79.3%)
Paid employment								
No	49	(29.3%)	47	(26.7%)	58	(34.3%)	154	(30.1%)
Yes	118	(70.7%)	129	(73.3%)	111	(65.7%)	358	(69.9%)
Living condition								
Alone	89	(53.3%)	91	(51.7%)	96	(56.8%)	276	(53.9%)
With someone	78	(46.7%)	85	(48.3%)	73	(43.2%)	236	(46.1%)

For peer review only

Table S3: Binary logistic regression on active interest to read more about vaccination

		Model 1: Overall sample					Model 2: Only White ethnic background					Model 3: Black, Asian, mixed or other ethnic backgound			
	(%)	OR	95% CI	aOR	95% CI	(%)	OR	95% CI	aOR	95% CI	(%)	OR	95% CI	aOR	95% CI
Condition															
Control	34.7	Ref.		Ref.		33.3	Ref.		Ref.		35.8	Ref.		Ref.	
Society	44.9	1.531	0.990 - 2.366	1.472	0.940 - 2.307	42.3	1.446	0.726 - 2.882	1.587	0.891 - 2.827	46.9	1.587	0.891 - 2.827	1.570	0.870 - 2.833
Family and friends	42.6	1.395	0.898 - 2.168	1.484	0.933 - 2.360	47.4	1.896	0.876 - 4.103	1.205	0.685 - 2.119	40.1	1.205	0.685 - 2.119	1.270	0.704 - 2.290
Vaccination status															
Invited	38.1	Ref.		Ref.		38.6	Ref.		Ref.		37.7	Ref.		Ref.	
Not invited	44.8	1.318	0.921 - 1.884	1.581	1.067 - 2.342*	42.8	1.238	0.701 - 2.185	1.456	0.770 - 2.754	45.4	1.372	0.865 - 2.176	1.621	0.979 - 2.685
Age															
18-24 years old	36.9	Ref.		Ref.		33.3	Ref.		Ref.		38.6	Ref.		Ref.	
25-34 years old	39.4	1.112	0.728 - 1.697	1.182	0.735 - 1.901	40.2	1.343	0.664 - 2.717	1.597	0.734 - 3.474	38.7	1.002	0.581 - 1.727	0.901	0.489 - 1.662
35-44 years old	50.8	1.764	0.973 - 3.200	1.722	0.889 - 3.333	53.8	2.333	0.672 - 8.101	2.726	0.707 - 10.503	50.0	1.590	0.802 - 3.150	1.501	0.693 - 3.252
45+ years old	45.3	1.416	0.782 - 2.565	1.915	0.986 - 3.718	48.3	1.867	0.726 - 4.796	2.415	0.839 - 6.951	42.9	1.192	0.546 - 2.601	1.351	0.559 - 3.266
Gender															
Male	42.3	Ref.		Ref.		39.8	Ref.		Ref.		44.5	Ref.		Ref.	
Female	39.6	0.895	0.626 - 1.278	0.898	0.617 - 1.308	41.6	1.076	0.616 - 1.882	1.113	0.614 - 2.019	38.6	0.782	0.490 - 1.249	0.809	0.494 - 1.325
Non-binary	40.0	0.908	0.149 - 5.541	0.657	0.102 - 4.213	33.3	0.756	0.066 - 8.611	0.547	0.045 - 6.582	50.0	1.245	0.076 - 20.382	1.112	0.065 - 19.070
Ethnicity															
White	40.6	Ref.		Ref.					.			.		.	
Mixed	40.6	1.000	0.574 - 1.742	0.908	0.510 - 1.616										
Asian	52.0	1.586	0.981 - 2.565	1.468	0.884 - 2.440										
Black	34.9	0.787	0.495 - 1.250	0.742	0.456 - 1.209										
Arab or other	15.4	0.266	0.058 - 1.232	0.280	0.059 - 1.323										
A-levels															
No	33.0	Ref.		Ref.		32.7	Ref.		Ref.		33.3	Ref.		Ref.	
Yes	42.9	1.521	0.970 - 2.386	1.692	1.042 - 2.746*	43.2	1.568	0.809 - 3.035	2.253	1.072 - 4.735*	42.6	1.486	0.801 - 2.759	1.611	0.827 - 3.140
Living condition															
Alone	39.1	Ref.		Ref.		41.1	Ref.		Ref.		38.1	Ref.		Ref.	
With somebody	42.8	1.164	0.817 - 1.657	0.973	0.652 - 1.452	40.2	0.964	0.553 - 1.683	0.873	0.476 - 1.603	45.2	1.337	0.841 - 2.125	1.196	0.700 - 2.041
Paid employment															
No	38.3	Ref.		Ref.		49.1	Ref.		Ref.		32.0	Ref.		Ref.	
Yes	41.9	1.161	0.789 - 1.710	1.107	0.727 - 1.686	37.3	0.617	0.333 - 1.142	0.606	0.310 - 1.183	45.5	1.756	1.058 - 2.914*	1.627	0.947 - 2.795
N		512		512			207		207			305		305	

* $p<0.05$; ** $p<0.01$

For peer review only

Table S4: Ordered logistic regression on perceived importance of vaccination [1;5]

	Model 1: Overall sample				Model 2: Only White ethnic background				Model 3: Black, Asian, mixed or other ethnic backgound			
	OR	95% CI	aOR	95% CI	OR	95% CI	aOR	95% CI	OR	95% CI	aOR	95% CI
Condition												
Control	Ref.		Ref.		Ref.		Ref.		Ref.		Ref.	
Society	0.948	0.649 - 1.386	0.897	0.608 - 1.325	1.493	0.836 - 2.668	1.458	0.794 - 2.677	0.647	0.391 - 1.072	0.627	0.376 - 1.047
Family and friends	0.931	0.639 - 1.357	1.042	0.706 - 1.539	1.271	0.692 - 2.334	1.833	0.961 - 3.496	0.751	0.462 - 1.222	0.829	0.502 - 1.369
Vaccination status												
Invited	Ref.		Ref.		Ref.		Ref.		Ref.		Ref.	
Not invited	1.317	0.964 - 1.799	1.200	0.859 - 1.677	0.919	0.562 - 1.503	0.563	0.321 - 0.990*	1.728	1.149 - 2.599**	1.774	1.151 - 2.733**
Age												
18-24 years old	Ref.		Ref.		Ref.		Ref.		Ref.		Ref.	
25-34 years old	0.851	0.592 - 1.224	0.813	0.547 - 1.208	0.953	0.527 - 1.724	0.754	0.397 - 1.432	0.703	0.439 - 1.126	0.690	0.411 - 1.159
35-44 years old	0.754	0.451 - 1.260	0.689	0.393 - 1.208	0.495	0.180 - 1.362	0.364	0.119 - 1.112	0.905	0.491 - 1.669	0.905	0.463 - 1.769
45+ years old	0.401	0.238 - 0.675**	0.428	0.240 - 0.761**	0.321	0.143 - 0.719**	0.265	0.105 - 0.665**	0.478	0.237 - 0.965*	0.501	0.230 - 1.094
Gender												
Male	Ref.		Ref.		Ref.		Ref.		Ref.		Ref.	
Female	0.557	0.405 - 0.765**	0.537	0.387 - 0.745**	0.411	0.249 - 0.680**	0.375	0.223 - 0.631**	0.715	0.472 - 1.085	0.707	0.459 - 1.090
Non-binary	0.552	0.130 - 2.341	0.509	0.118 - 2.205	0.857	0.154 - 4.754	1.124	0.182 - 6.955	0.163	0.014 - 1.941	0.153	0.012 - 1.891
Ethnicity												
White	Ref.		Ref.				.		.		.	
Mixed	0.701	0.433 - 1.137	0.684	0.416 - 1.125								
Asian	1.132	0.740 - 1.731	1.100	0.707 - 1.710								
Black	0.718	0.482 - 1.071	0.633	0.417 - 0.960*								
Arab or other	0.693	0.259 - 1.854	0.707	0.253 - 1.975								
A-levels												
No	Ref.		Ref.		Ref.		Ref.		Ref.		Ref.	
Yes	1.741	1.188 - 2.549**	1.793	1.190 - 2.701**	2.221	1.277 - 3.865**	2.301	1.260 - 4.204**	1.435	0.840 - 2.453	1.466	0.827 - 2.599
Living condition												
Alone	Ref.		Ref.		Ref.		Ref.		Ref.		Ref.	
With somebody	1.036	0.758 - 1.416	1.088	0.770 - 1.536	0.908	0.558 - 1.478	0.811	0.483 - 1.360	1.117	0.742 - 1.683	1.415	0.883 - 2.266
Paid employment												
No	Ref.		Ref.		Ref.		Ref.		Ref.		Ref.	
Yes	1.263	0.906 - 1.761	1.120	0.788 - 1.590	1.545	0.910 - 2.625	1.252	0.714 - 2.196	1.093	0.711 - 1.679	1.076	0.680 - 1.702
N	512		512		207		207		305		305	

* *p*<0.05; ** *p*<0.01

For peer review only

Table S5: Ordered logistic regression on expected uptake of vaccination [1;5]

	Model 1: Overall sample				Model 2: Only White ethnic background				Model 3: Black, Asian, mixed or other ethnic backgound			
	OR	95% CI	aOR	95% CI	OR	95% CI	aOR	95% CI	OR	95% CI	aOR	95% CI
Condition												
Control	Ref.		Ref.		Ref.		Ref.		Ref.		Ref.	
Society	1.036	0.696 - 1.540	1.048	0.700 - 1.571	1.260	0.694 - 2.288	1.443	0.779 - 2.674	0.856	0.501 - 1.461	0.863	0.504 - 1.480
Family and friends	0.864	0.585 - 1.276	0.885	0.592 - 1.324	0.852	0.443 - 1.636	1.000	0.508 - 1.970	0.874	0.535 - 1.429	0.823	0.496 - 1.365
Vaccination status												
Invited	Ref.		Ref.		Ref.		Ref.		Ref.		Ref.	
Not invited	0.598	0.431 - 0.830**	0.640	0.451 - 0.908*	0.491	0.290 - 0.831**	0.513	0.290 - 0.909*	0.675	0.443 - 1.029	0.720	0.459 - 1.129
Age												
18-24 years old	Ref.		Ref.		Ref.		Ref.		Ref.		Ref.	
25-34 years old	1.118	0.765 - 1.635	1.017	0.666 - 1.553	1.375	0.734 - 2.574	1.106	0.562 - 2.179	0.915	0.561 - 1.493	0.988	0.573 - 1.702
35-44 years old	1.405	0.818 - 2.415	1.324	0.737 - 2.380	1.283	0.399 - 4.120	1.431	0.444 - 4.608	1.442	0.778 - 2.673	1.486	0.748 - 2.954
45+ years old	1.472	0.849 - 2.553	1.332	0.727 - 2.441	1.479	0.622 - 3.517	1.197	0.464 - 3.088	1.463	0.708 - 3.020	1.557	0.694 - 3.493
Gender												
Male	Ref.		Ref.		Ref.		Ref.		Ref.		Ref.	
Female	0.839	0.606 - 1.162	0.802	0.573 - 1.123	0.840	0.503 - 1.402	0.838	0.495 - 1.418	0.875	0.570 - 1.345	0.830	0.533 - 1.292
Non-binary	0.256	0.056 - 1.171	0.289	0.060 - 1.401	0.170	0.021 - 1.342	0.231	0.029 - 1.867	0.406	0.048 - 3.450	0.469	0.050 - 4.374
Ethnicity												
White	Ref.		Ref.				.		.		.	
Mixed	0.839	0.507 - 1.390	0.842	0.502 - 1.412								
Asian	1.067	0.686 - 1.659	1.035	0.649 - 1.650								
Black	0.653	0.432 - 0.989*	0.653	0.425 - 1.005								
Arab or other	0.838	0.311 - 2.258	0.896	0.324 - 2.480								
A-levels												
No	Ref.		Ref.		Ref.		Ref.		Ref.		Ref.	
Yes	1.312	0.873 - 1.973	1.272	0.827 - 1.958	1.408	0.771 - 2.571	1.249	0.661 - 2.362	1.306	0.744 - 2.292	1.363	0.741 - 2.505
Living condition												
Alone	Ref.		Ref.		Ref.		Ref.		Ref.		Ref.	
With somebody	0.995	0.722 - 1.370	0.828	0.581 - 1.179	1.019	0.612 - 1.696	0.858	0.501 - 1.469	0.942	0.620 - 1.432	0.852	0.533 - 1.362
Paid employment												
No	Ref.		Ref.		Ref.		Ref.		Ref.		Ref.	
Yes	1.158	0.815 - 1.644	1.102	0.761 - 1.596	1.686	0.950 - 2.990	1.555	0.859 - 2.812	0.914	0.585 - 1.427	0.876	0.545 - 1.408
N	512		512		207		207		305		305	

* p<0.05; ** p<0.01

For peer review only

Table S6. Adjusted logistic regression with interaction terms for condition and ethnicity on intentions, expectations and perceived social importance and active interest (N=512)

	Model 1: Intentions [1;8]		Model 2: Expectations [1;5]		Model 3: Social importance [1;5]		Model 4: Active interest [0;1]	
	aOR	95% CI	aOR	95% CI	aOR	95% CI	aOR	95% CI
Condition and ethnicity								
Control – White	Ref.		Ref.		Ref.		Ref.	
Control – Ethnic minoties	1.304	0.747 - 2.277	1.019	0.570 - 1.824	1.264	0.721 - 2.215	1.041	0.536 - 2.020
Society – White	1.984	1.090 - 3.614*	1.411	0.776 - 2.567	1.529	0.826 - 2.829	1.440	0.730 - 2.839
Society – Ethnic minorities	1.301	0.748 - 2.265	0.873	0.485 - 1.574	0.842	0.480 - 1.476	1.661	0.870 - 3.172
Family and friends - White	1.681	0.886 - 3.189	0.964	0.501 - 1.857	1.568	0.825 - 2.981	1.931	0.921 - 4.047
Family and friends – Ethnic minority	1.419	0.818 - 2.462	0.836	0.476 - 1.465	1.026	0.589 - 1.787	1.287	0.673 - 2.460
Vaccination status								
Invited	Ref.		Ref.		Ref.		Ref.	
Not invited	1.135	0.819 - 1.574	0.625	0.440 - 0.887**	1.164	0.833 - 1.627	1.515	1.026 - 2.237*
Age								
18-24 years old	Ref.		Ref.		Ref.		Ref.	
25-34 years old	0.951	0.647 - 1.397	1.025	0.674 - 1.559	0.804	0.543 - 1.191	1.152	0.721 - 1.840
35-44 years old	0.844	0.490 - 1.454	1.404	0.784 - 2.514	0.749	0.431 - 1.302	1.918	0.999 - 3.682
45+ years old	0.293	0.164 - 0.523**	1.346	0.737 - 2.460	0.428	0.241 - 0.761**	1.824	0.943 - 3.526
Gender								
Male	Ref.		Ref.		Ref.		Ref.	
Female	0.677	0.491 - 0.933*	0.829	0.593 - 1.158	0.546	0.394 - 0.757**	0.908	0.626 - 1.317
Non-binary	0.360	0.082 - 1.577	0.281	0.059 - 1.340	0.457	0.107 - 1.951	0.715	0.112 - 4.552
A-levels								
No	Ref.		Ref.		Ref.		Ref.	
Yes	1.629	1.079 - 2.458*	1.264	0.820 - 1.948	1.822	1.210 - 2.745**	1.783	1.097 - 2.899*
Living condition								
Alone	Ref.		Ref.		Ref.		Ref.	
With somebody	1.263	0.902 - 1.768	0.856	0.602 - 1.216	1.127	0.800 - 1.588	1.012	0.682 - 1.503
Paid employment								
No	Ref.		Ref.		Ref.		Ref.	
Yes	1.145	0.809 - 1.619	1.088	0.753 - 1.572	1.112	0.784 - 1.578	1.101	0.728 - 1.666
N	512		512		512		512	

* p<0.05; ** p<0.01

For peer review only

BMJ Open

The effect of communicating community immunity on COVID-19 vaccine-hesitant people from ethnically diverse backgrounds: an experimental vignette study in the UK

Journal:	*BMJ Open*
Manuscript ID	bmjopen-2022-065804.R1
Article Type:	Original research
Date Submitted by the Author:	01-Oct-2022
Complete List of Authors:	Stoffel, Sandro T.; UCL, Research Department of Behavioural Science and Health; University of Basel<br>Kaushal, Aradhna; University College London, Behavioural Science and Health<br>Grimani, Aikaterini; University of Warwick, Warwick Business School<br>von Wagner, Christian; University College London Department of Behavioural Science and Health<br>Sniehotta, Falko F.; Newcastle University; Heidelberg University, Department of Public Health, Preventive and Social Medicine<br>Vlaev, Ivo; Warwick Business School
**Primary Subject Heading**:	Public health
Secondary Subject Heading:	Infectious diseases
Keywords:	COVID-19, PUBLIC HEALTH, INFECTIOUS DISEASES

SCHOLARONE™
Manuscripts

I, the Submitting Author has the right to grant and does grant on behalf of all authors of the Work (as defined in the below author licence), an exclusive licence and/or a non-exclusive licence for contributions from authors who are: i) UK Crown employees; ii) where BMJ has agreed a CC-BY licence shall apply, and/or iii) in accordance with the terms applicable for US Federal Government officers or employees acting as part of their official duties; on a worldwide, perpetual, irrevocable, royalty-free basis to BMJ Publishing Group Ltd ("BMJ") its licensees and where the relevant Journal is co-owned by BMJ to the co-owners of the Journal, to publish the Work in this journal and any other BMJ products and to exploit all rights, as set out in our licence.

The Submitting Author accepts and understands that any supply made under these terms is made by BMJ to the Submitting Author unless you are acting as an employee on behalf of your employer or a postgraduate student of an affiliated institution which is paying any applicable article publishing charge ("APC") for Open Access articles. Where the Submitting Author wishes to make the Work available on an Open Access basis (and intends to pay the relevant APC), the terms of reuse of such Open Access shall be governed by a Creative Commons licence – details of these licences and which Creative Commons licence will apply to this Work are set out in our licence referred to above.

Other than as permitted in any relevant BMJ Author's Self Archiving Policies, I confirm this Work has not been accepted for publication elsewhere, is not being considered for publication elsewhere and does not duplicate material already published. I confirm all authors consent to publication of this Work and authorise the granting of this licence.

For peer review only

The effect of communicating community immunity on COVID-19 vaccine-hesitant people from ethnically diverse backgrounds: an experimental vignette study in the UK

Sandro T. Stoffel[1,2], Aradhna Kaushal[1], Aikaterini Grimani[3], Christian von Wagner[1], Falko F. Sniehotta[4,5], Ivo Vlaev[3]

Correspondence to: Dr Aikaterini Grimani; aikaterini.grimani@wbs.ac.uk

University of Warwick, Warwick Business School, Scarman Rd, CV4 7AL, Coventry, UK

1. Research Department of Behavioural Science and Health, UCL, London, UK
2. Institute for Pharmaceutical Medicine, University of Basel, Basel; Switzerland
3. NIHR Policy Research Unit in Behavioural Science - Behavioural Science Group, Warwick Business School, University of Warwick, Coventry, UK
4. NIHR Policy Research Unit in Behavioural Science - Population Health Sciences Institute, Faculty of Medical Sciences, Newcastle University, Newcastle upon Tyne, UK.
5. Department of Public Health, Preventive and Social Medicine, Centre for Preventive Medicine and Digital Health Baden-Wuerttemberg, Heidelberg University, Mannheim, Germany

Words: 3273

Tables: 2

Figures: 1

For peer review only

Abstract

Objectives: Achieving high vaccination coverage is vital to the efforts of curbing the impact of the COVID-19 pandemic on public health and society. This study tested whether communicating the social benefit through community protection for friends and family members vs. overall society, affects vaccination intention and perception among a sample enriched with respondents from black and ethnic minority backgrounds.

Design: A web-based experimental survey was conducted. Eligible participants were individually randomised, with equal probability, to one of the three experimental vignettes.

Setting: England

Participants: We recruited 512 (212 white, 300 ethnically diverse) vaccine-hesitant members from an online panel.

Primary and secondary outcome measures: The primary outcome was the intention to get vaccinated against COVID-19. The secondary outcome consisted of a behavioural measure in the form of active interest in reading more about the coronavirus (COVID-19) vaccine. Additional measures included the perceived importance and expected uptake in others, as well as the attitudes towards vaccination.

Results: Logistic regression models did not show an effect of the messages on intentions for the overall sample (*society*: adjusted odds ratio (aOR): 128, 95% confidence interval (95%CI) 0.88-1.88 and *friends and family*: aOR 1.32, 95%CI 0.89-1.94). The role of vaccination in achieving community immunity yielded higher vaccination intentions among study participants with White ethnic background (*society*: aOR: 1.94, 95%CI 1.07-3.51 and *friends and family*: aOR 2.07, 95%CI 1.08-3.96), but not among respondents from ethnically diverse backgrounds (*society*: aOR: 0.95, 95%CI 0.58-1.58 and *friends and family*: aOR 1.06, 95%CI 0.64-1.73). The messages,

For peer review only

however, did not affect the perceived importance of the vaccine, expected vaccination uptake and active interest in reading more about the vaccine.

Conclusions: Thus, although highlighting the social benefits of COVID-19 vaccinations can increase intentions among vaccine non-intenders, they are unlikely to address barriers among ethnically diverse communities.

Strengths and limitations of this study

- This is one of the first experimental studies that investigated how the framing of the beneficiaries of community immunity influences vaccination intentions.
- The study purposely recruited as many study participants from ethnic minorities as possible.
- The study had a small analytical sample due to the difficulties in recruiting vaccine-hesitant study participants in the ongoing vaccination programme, limiting statistical power.
- We had to combine all ethnic minorities so that nothing can be said about the impact of the messages on different ethnic minorities.
- The study was conducted in May 2021, when the health policies during the pandemic were rapidly changing, limiting so the generalisability of the results.

For peer review only

Introduction

The SARS-CoV-2 (COVID-19) pandemic has led to over 85 thousand excess deaths in England and Wales and almost 6 million reported COVID-19 deaths between Jan 1, 2020, and Dec 31, 2021 [1, 2]. The success of the coronavirus (COVID-19) vaccination program depends on high levels of coverage, around 80%, to achieve community immunity [3]. This is increasingly important as vaccine effectiveness wanes over time and may not necessarily confer protection against the new COVID-19 variants, requiring booster vaccinations to maintain a high level of coverage [4]. However, some subgroups of the population are more likely to be vaccine hesitant with uptake and intention varying by gender, age, education, employment status, deprivation, and ethnicity [5-7].

Data from the Office for National Statistics show higher rates of vaccine hesitancy in younger age groups, women, people working in hospitality, personal services or transport sectors, people living in more deprived areas, and people from ethnic minority groups, particularly Black/Black British and Pakistani/Bangladeshi ethnic groups [6, 8, 9]. This is particularly concerning as many of these groups are at increased risk of contracting COVID-19, hospitalisation and death from COVID-19 [10-12]. Reasons for vaccine hesitancy include worry about the side effects, worry about the long-term effects on health, waiting to see how effective vaccines are, and concerns about safety [13].

A key method to promote vaccine uptake among individuals is to design public health messages to promote collective goals and use targeted messages as a mechanism to achieve the same wider collective aim, e.g., save the NHS, protect lives, promote awareness of symptoms and control measures [14-18]. These ‘Protect each other’ messages highlight the benefits of protective behaviours for the group and its most

For peer review only

vulnerable members by promoting care for others rather than individual self-interest [19]. In addition, enlisting trusted voices has been shown to make public health messages more effective in changing behaviour during epidemics [20, 21].

While experimental studies on social preferences have shown that individuals are motivated by the well-being of others, individuals may hold social identities at various levels of abstraction, ranging from concrete groups of individuals (e.g., own friends and family) to broader categories of individuals such as citizens of their country [22, 23]. Studies on social preferences have shown that individuals exhibit more prosocial behaviour if they know more about the potential beneficiaries, such as their social belonging [23-26]. Thus, prosocial behaviour is negatively related to social distance; e.g. whether beneficiaries are close friends or not [27-29]. As such, individuals' intentions to get immunized for the benefit of others may depend on the social distance to the beneficiaries of community immunity. A recent experimental study found that people in England had higher intentions to get vaccinated against a hypothetical flu if they were told about the social benefit of vaccination for their close social environment, such as friends and family. Communicating the social benefit for the overall society, however, did not increase vaccination intentions [30]. Furthermore, as the cultural background can influence the perception of social benefits, communicating the concept of herd immunity can increase vaccination intentions among cultures that focus on collective benefits. Prosocial nudges can thus help to close these immunity gaps (22).

The primary aim of the current research was to explore how the framing of community immunity influences vaccination intentions among White and ethnic minorities. For this, we conducted a web-based experiment and recruited vaccine-hesitant individuals with a White ethnicity and an ethnic minority background. Specifically, we investigated whether the definition of the beneficiaries of community immunity affects vaccination

For peer review only

intentions, expecting that highlighting the indirect effect of immunization for the close social environment would increase intentions to get a coronavirus (COVID-19) vaccine. In line with previous research, we expect that the information about community immunity to be more effective in increasing vaccination intentions, if it mentions close social environments instead of overall society, as the definition of the beneficiaries influences social preferences [30, 31]. Additionally, the study also investigated whether communicating community immunity influences the perception of the vaccine in terms of social importance and expected uptake of peers.

Methods

General procedure. A web-based experimental survey was programmed in Qualtrics to assess how the framing of community immunity influences vaccination intentions. The survey was conducted in May 2021 and featured a sample of adult men and women who were invited by a survey vendor (Dynata) to take part in an online survey on COVID-19 vaccination. Only study participants who completed the survey received a small financial incentive from the survey vendor, which was defined by the length of the questionnaire.

Survey design. At the start of the survey, study participants were asked to give explicit consent for their data to be used and published as part of this research project before they could continue in the survey. A first screener question on screening behaviour was used to recruit individuals who had either been invited for the vaccination but declined it or who have not been invited yet but did not intend to get vaccinated when invited. The question was adapted from the NIHR Policy Research Unit Behavioural Sciences questionnaire on vaccination intentions. Participants were told that they would have the vaccine once it is available to them, with the response options; strongly disagree, disagree, neither agree nor disagree, agree and strongly agree [32].

For peer review only

Individuals who disagreed or neither agreed nor disagreed were then asked about their ethnicity. As vaccine hesitancy was identified as being common among ethnic minority groups, a quota was employed on White ethnicity to recruit no more than 215 respondents from a White ethnic background and at least 300 study participants with an ethnic minority background so that meaningful differences could be explored. Individuals who were filtered out from the survey were redirected to the study briefing and final survey page where they were thanked for their participation (see Figure 1).

[Figure 1 here]

Experimental conditions. Eligible individuals were individually randomised, with equal probability, to one of three experimental vignettes with different versions of the COVID-19 vaccination invitation letter. Each participant was told that the vaccination would be free and consisted of two separate shots that would be administered in a vaccination centre. The type of vaccine was not mentioned in the vignettes. The last sentence of the vignette featured the experimental manipulation with the description of the beneficiaries of the vaccination (see Table 1).

For peer review only

Table 1. Behavioural messages tested in the experiment

**Condition**	**Final paragraph of the vignette**
Control	Get vaccinated to protect yourself from Covid-19.
Society	Get vaccinated to protect yourself and the vulnerable people in Great Britain from Covid-19.
Family and friends	Get vaccinated to protect yourself, your community, and your vulnerable friends and family members such as your children, your parents or your grandparents from the coronavirus (COVID-19).

Ethics approval and preregistration. The research project received ethical approval from the University of Warwick's Humanities and Social Sciences Research Ethics Committee (HSSREC 142/20-21). The design and analysis plan for the experiment had been preregistered on Open Science Framework (OSF; https://osf.io/k4jyv/?view_only=08694ea40f994671b2dd899ae656f34e) before any data was collected. Questionnaire (see supplementary file_questionnaire), analysis scripts and an abridged dataset containing all primary study variables can also be found on OSF.

Outcome measures

Vaccination intentions. The primary outcome was the intention to get vaccinated against COVID-19. This was measured using the question: "Would you get vaccinated against the coronavirus?" with responses on a partially labelled eight-point scale, where 1 means that one will definitely not get vaccinated and 8 indicates that one will definitely get vaccinated [30]. The response options to these questions were taken from previous studies [33, 34]. Individuals who stated low vaccination intentions (below 5) were asked to indicate reasons for not wanting to get vaccinated (see Supplementary Table S1 for answers).

Active interest. The secondary outcome consisted of a behavioural measure in the form of active interest in reading more about the coronavirus (COVID-19) vaccine [35,

For peer review only

36]. Following the experimental manipulation towards the end of the survey, participants were asked whether they would like to ‘read’ or ‘skip’ information about the vaccination procedure before completing the survey. Those who indicated that they would like to skip it were sent to the end of the questionnaire with the socio-demographic question, while those who wanted to read the information were presented additional information about the vaccination from the official website of the publicly funded national health service of England (NHS). Choosing to read the information was interpreted as an active interest in reading more health information. Engagement with the information was measured with four comprehension questions.

Perceived importance and expected uptake in others. To explore the impact of the experimental manipulation on the perceived importance of the immunization programme, we included the question. “Do you believe that getting vaccinated against Covid-19 is socially important?”. Additionally, we asked whether they thought that most people would get immunized with the question “Do you think that most people will get vaccinated?” Both items featured five-point fully-labelled Likert scale response options [30].

Attitudes towards vaccination. Nine additional questions from the 5C measure of psychological antecedents of vaccination were included as further outcomes [37]. The questions featured 7-point fully-labelled Likert scales [0;6] and were combined into three constructs on confidence in vaccination and public authorities, complacency and perceived collective responsibility with scores between 0 and 18 each.

Sample size calculation and statistical analysis

The sample size for the experiment was based on estimates from a previous study [30]. A sample size calculation suggested minimum group sizes of 170 participants per group (510 participants in total) [30, 38]. We used A Kruskal-Wallis tests and

For peer review only

multivariable ordered and binary logistic regressions adjusting for baseline vaccination invitation status and sociodemographic variables to investigate the effect of the experimental conditions on vaccination intentions and active interest. The regressions were run on the whole sample and the two ethnic subgroups; White and BAME (Black, Asian and minority ethnic) which describes non-white ethnicities. The statistical analysis was conducted with Stata/IC version 16.0 (StataCorp LP, College Station, TX).

Patient and public involvement (PPI)

There was no patient or public involvement in the design, conduct and reporting of this research.

Results

Study sample. The analytical sample comprised 512 individuals who had not yet been invited for a COVID-19 vaccination appointment but were planning not to accept the invitation (N=302; 59.0%) or have been invited and had declined the vaccination (N=210, 41.0%) More than half of the study participants were from an ethnic minority (excluding white minorities) group (N=307, 59.6%), female (N=285, 55.7%) and between 25 and 34 years old (N=236, 46.1%). There were no statistically significant differences in sociodemographic characteristics, indicating that there were no imbalances due to levels of drop-out varying among the three experimental conditions (see Table S2 in the supplementary file for the characteristics of the study sample).

Vaccination intentions. The results of the ordered logistic regression, reported in Table 2, showed that, compared with the control condition, neither the society nor the family and friends message yielded higher vaccination intentions (see Figure S1 in the supplementary file for the distribution). Looking at White and ethnic minority subgroups separately reveals that the behavioural messages were associated with higher

For peer review only

vaccination intentions in the former but not in the latter group (adjusted odds ratio (aOR): 1.94; 95% confidence interval (CI): 1.07–3.51 and aOR: 2.07; 95% CI: 1.08–3.96).

Active interest. The experimental manipulations did not influence interest in reading more about the vaccination process (see Supplementary Table S3 for the regression results). Independently from the experimental condition, around one-third of the respondents stated that they wanted to read more (34.7–44.9%, $\chi^2(2, N=512) = 3.991$, $p=0.136$). A Kruskal-Wallis test did not reveal any differences in vaccination knowledge across the conditions ($\chi2=0.655$, $p=0.721$, $df=2$). Most participants (45.9%) who read the additional information answered 2 out of 4 comprehension questions correctly.

Perceived importance and expected uptake in others. Only a minority of participants stated that they perceived the vaccinations as not important (N=129, 25.2%) and most thought the vaccination to be moderately important (N=163, 31.8%). Across the three conditions, the majority (N=271, 52.9%) expected that more than 60% of their peers would get vaccinated. Importantly, the experimental manipulations did not affect the perceived importance of the vaccination nor the expected uptake (see Supplementary Table S4, S5 and S6 for the regression results and Figures S2 and S3 for the distributions).

Attitude towards vaccination. The median values for confidence in vaccination and public authorities was only 9 out of 18 (SD 4.84) and did not vary between experimental conditions ($\chi^2 = 0.05$, $p = 0.974$, $df = 2$). Similarly, complacency, the feeling that vaccination is not necessary was also low relatively with a median of 8 out of 18 (SD 4.51) and also no variation between experimental conditions ($\chi^2 = 2.48$, $p = 0.290$, $df = 2$). The median for perceived social responsibility was 11 out of 18 (SD 2.30) and

For peer review only

Table 2: Ordered logistic regression on vaccination intentions [1;8]

	Model 1: Overall sample					Model 2: Only White ethnic background					Model 3: Black, Asian, mixed or other ethnic backgrounds				
	Mean	OR	95% CI	aOR	95% CI	Mean	OR	95% CI	aOR	95% CI	Mean	OR	95% CI	aOR	95% CI
Condition															
Control	3.60	Ref.		Ref.		3.42	Ref.		Ref.		3.74	Ref.		Ref.	
Society	3.98	1.362	0.936 - 1.981	1.284	0.876 - 1.880	4.27	1.965	1.103 - 3.498*	1.937	1.069 - 3.512*	3.74	0.999	0.609 - 1.638	0.952	0.577 - 1.571
Family and friends	3.86	1.200	0.826 - 1.744	1.317	0.893 - 1.942	3.89	1.430	0.779 - 2.627	2.066	1.077 - 3.962*	3.84	1.030	0.638 - 1.663	1.056	0.644 - 1.732
Vaccination status															
Invited	3.70	Ref.		Ref.		3.85	Ref.		Ref.		3.59	Ref.		Ref.	
Not invited	3.98	1.263	0.929 - 1.718	1.192	0.859 - 1.654	3.90	1.035	0.636 - 1.683	0.678	0.394 - 1.169	4.02	1.450	0.974 - 2.161	1.516	0.993 - 2.315
Age															
18-24 years old	3.97	Ref.		Ref.		4.42	Ref.		Ref.		3.75	Ref.		Ref.	
25-34 years old	4.04	1.075	0.753 - 1.536	0.932	0.632 - 1.375	4.15	0.839	0.465 - 1.512	0.694	0.370 - 1.304	3.94	1.179	0.743 - 1.872	0.968	0.584 - 1.604
35-44 years old	3.84	0.910	0.549 - 1.509	0.743	0.426 - 1.296	2.93	0.346	0.120 - 0.996*	0.289	0.089 - 0.945*	4.08	1.311	0.726 - 2.366	1.085	0.567 - 2.076
45+ years old	2.58	0.297	0.173 - 0.508**	0.289	0.161 - 0.519**	2.24	0.182	0.077 - 0.430**	0.163	0.064 - 0.416**	2.86	0.423	0.209 - 0.855*	0.347	0.159 - 0.755**
Gender															
Male	4.08	Ref.		Ref.		4.25	Ref.		Ref.		3.93	Ref.		Ref.	
Female	3.62	0.697	0.511 - 0.953*	0.653	0.473 - 0.901**	3.50	0.562	0.344 - 0.917*	0.528	0.319 - 0.876*	3.68	0.807	0.536 - 1.215	0.768	0.503 - 1.174
Non-binary	3.00	0.480	0.120 - 1.927	0.358	0.082 - 1.553	3.00	0.422	0.063 - 2.802	0.394	0.045 - 3.447	3.00	0.512	0.069 - 3.820	0.401	0.050 - 3.185
Ethnicity															
White	3.87	Ref.		Ref.					.			.		.	
Mixed	3.46	0.741	0.461 - 1.192	0.687	0.419 - 1.126										
Asian	4.42	1.509	0.998 - 2.281	1.477	0.959 - 2.277										
Black	3.43	0.713	0.479 - 1.061	0.673	0.445 - 1.018										
Arab or other	3.77	0.904	0.324 - 2.525	1.017	0.338 - 3.059										
A-levels															
No	3.33	Ref.		Ref.		3.13	Ref.		Ref.		3.52	Ref.		Ref.	
Yes	3.94	1.664	1.132 - 2.444**	1.609	1.064 - 2.431*	4.12	2.033	1.167 - 3.542*	2.095	1.143 - 3.838*	3.83	1.323	0.769 - 2.275	1.285	0.724 - 2.283
Living condition															
Alone	3.72	Ref.		Ref.		4.00	Ref.		Ref.		3.57	Ref.		Ref.	
With somebody	3.92	1.170	0.861 - 1.591	1.207	0.861 - 1.693	3.76	0.840	0.519 - 1.361	0.806	0.485 - 1.341	4.07	1.533	1.025 - 2.293*	1.815	1.143 - 2.884*
Paid employment															
No	3.52	Ref.		Ref.		3.43	Ref.		Ref.		3.57	Ref.		Ref.	
Yes	3.94	1.366	0.984 - 1.895	1.174	0.828 - 1.664	4.03	1.521	0.896 - 2.580	1.229	0.700 - 2.157	3.86	1.273	0.837 - 1.935	1.064	0.678 - 1.671
N		512		512			207		207			305		305	

* $p<0.05$; ** $p<0.01$

For peer review only

was also not influenced by the experimental manipulations (χ^2 = 0.78, p = 0.679, df = 2). See Figure S4 in the supplementary file for the distribution of the attitude scores.

Reasons for vaccination hesitancy. Among the 306 study participants who did not intend to be vaccinated, fear of side effects and doubts about the protective nature of the vaccine were the most frequently mentioned reasons to not get vaccinated (66%) followed by not believing that the vaccine offers protection (38%). See Table S1 in the supplementary file for a full list of the stated reasons.

General discussion

This is one of the first studies to investigate how the framing of the beneficiaries of community immunity influences vaccination intentions [30, 31]. In a web-based experimental study, we tested whether mentioning the social benefit of vaccination through community protection for friends and family members or overall society affects COVID-19 vaccination behaviour among white and ethnic minorities. Contrary to a previous study (Stoffel and Herrmann, 2021), adding this information only affected vaccination intentions among respondents from a White Ethnic background. This lack of effect among respondents from ethnic minorities may be due to previously reported high levels of medical mistrust in ethnic minority groups which was found to mediate participation of Black participants in COVID-19 vaccine trials and uptake more generally [39]. Future research could explore this further by testing messages relating to vaccine safety and efficacy.

Limitations of the experimental study

Our study has several limitations which call for follow-up research. First, we only had a small analytical sample due to the difficulties in recruiting vaccine-hesitant study participants in the ongoing vaccination programme, limiting statistical power. Second, the study featured study samples from an online panel, which may limit the

For peer review only

generalisability of the results. Third, we had to combine all ethnic minorities so that nothing can be said about the impact of the messages on different ethnic minorities. Fourth, we assessed vaccination intentions and willingness to read more about the vaccine. Therefore, the utility of communicating community immunity in changing vaccination behaviour cannot be determined as intentions do not necessarily translate to behaviours [40, 41]. Thus, additional strategies may be required to build on motivational changes to increase vaccination uptake, such as implementation intentions [42]. Moreover, the study was conducted in May 2021, the health policies during the pandemic were rapidly changing, limiting so the generalisability of the results. A final limitation is that the survey did not include questions about the perceived risk of the responder and their family. It is possible that the different effects of the messages on White and ethnic minorities could have been caused by differences in perceived risk. A recent study from the US showed that racial and ethnic minorities are more likely to perceive COVID-19 as a major threat to the population and their individual health [43].

Conclusion

This study tested how the framing of community immunity affects intentions to get the COVID-19 vaccine among vaccine-hesitant individuals. Focusing on ethnic minority groups, the study investigated whether behavioural messages appealing to get vaccinated to protect oneself, family members and friends or the overall society increased vaccination intentions and interest in reading more about the vaccine. As the behavioural messages failed to affect the vaccination behaviour of vaccine-hesitant participants, future studies should develop interventions that are specifically targeted at reducing fear about side effects and concerns about the protective nature of the COVID-19 vaccine to achieve the highest possible level of vaccination uptake.

For peer review only

Contributorship statement

Conceptualisation: SS, CVW, AK, AG, FFS, IV; Data curation: AK, SS; Formal analysis, AK, SS, IV; Investigation: SS, AG, CVW, AK, FFS, IV; Methodology: SS, AG, CVW, AK, FFS, IV; Supervision, FFS, IV; Writing – original draft: SS, AK; Writing – review & editing: SS, CVW, AK, AG, FFS, IV.

Competing Interests statement

The authors declare no conflict of interest.

Funding Statement

This project is funded by the National Institute for Health Research (NIHR) [Policy Research Program (Policy Research Unit in Behavioural Science PR-PRU1217-20501)]. The views expressed are those of the author(s) and not necessarily those of the NIHR or the Department of Health and Social Care.

Data availability statement

The data presented in this study are available upon reasonable request from the corresponding author.

Ethics approval

The research project received ethical approval from the University of Warwick's Humanities and Social Sciences Research Ethics Committee (HSSREC 142/20-21). Participants gave informed consent to participate in the study before taking part.

For peer review only

References

1. Islam, N., et al., *Excess deaths associated with covid-19 pandemic in 2020: age and sex disaggregated time series analysis in 29 high income countries.* bmj, 2021. **373**.

2. Wang, H., et al., *Estimating excess mortality due to the COVID-19 pandemic: a systematic analysis of COVID-19-related mortality, 2020–21.* The Lancet, 2022. **399**(10334): p. 1513-1536.

3. Mills, M., et al., *COVID-19 vaccine deployment: Behaviour, ethics, misinformation and policy strategies.* London: The Royal Society & The British Academy, 2020.

4. Feikin, D.R., et al., *Duration of effectiveness of vaccines against SARS-CoV-2 infection and COVID-19 disease: results of a systematic review and meta-regression.* The Lancet, 2022.

5. Martin, C.A., et al., *Association of demographic and occupational factors with SARS-CoV-2 vaccine uptake in a multi-ethnic UK healthcare workforce: a rapid real-world analysis.* MedRxiv, 2021.

6. Robertson, E., et al., *Predictors of COVID-19 vaccine hesitancy in the UK household longitudinal study.* Brain, behavior, and immunity, 2021. **94**: p. 41-50.

7. Neumann-Böhme, S., et al., *Once we have it, will we use it? A European survey on willingness to be vaccinated against COVID-19*. 2020, Springer. p. 977-982.

8. Lockyer, B., et al., *Understanding COVID-19 misinformation and vaccine hesitancy in context: Findings from a qualitative study involving citizens in Bradford, UK.* Health Expectations, 2021. **24**(4): p. 1158-1167.

9. Public Health England, *Disparities in the risk and outcomes of COVID-19*. 2020, Public Health England London.

10. Office for National Statistics. *Why have Black and South Asian people been hit hardest by COVID-19?* 2020 8th February 2021]; Available from: https://www.ons.gov.uk/peoplepopulationandcommunity/healthandsocialcare/conditionsanddiseases/articles/whyhaveblackandsouthasianpeoplebeenhithardestbycovid19/2020-12-14

11. Aldridge, R.W., et al., *Black, Asian and Minority Ethnic groups in England are at increased risk of death from COVID-19: indirect standardisation of NHS mortality data.* Wellcome open research, 2020. **5**.

12. Paul, E., A. Steptoe, and D. Fancourt, *The Lancet Regional Health—Europe Attitudes towards vaccines and intention to vaccinate against COVID-19: Implications for public health communications.* Lancet Reg. Health Eur, 2021. **1**.

For peer review only

13. Office for National Statistics. *Coronavirus and vaccine hesitancy, Great Britain: 13 January to 7 February 2021*. 2021 11th November 2021]; Available from: https://www.ons.gov.uk/peoplepopulationandcommunity/healthandsocialcare/healthandwellbeing/bulletins/coronavirusandvaccinehesitancygreatbritain/13januaryto7february2021.

14. Kelly, B.J. and R.C. Hornik, *Effects of framing health messages in terms of benefits to loved ones or others: An experimental study.* Health Communication, 2016. **31**(10): p. 1284-1290.

15. Li, M., et al., *Stimulating influenza vaccination via prosocial motives.* PloS one, 2016. **11**(7): p. e0159780.

16. Ghio, D., et al., *What influences people's responses to public health messages for managing risks and preventing infectious diseases? A rapid systematic review of the evidence and recommendations.* BMJ open, 2021. **11**(11): p. e048750.

17. Jordan, J.J., E. Yoeli, and D.G. Rand, *Don't get it or don't spread it: Comparing self-interested versus prosocial motivations for COVID-19 prevention behaviors.* Scientific reports, 2021. **11**(1): p. 1-17.

18. Everett, J.A., et al., *The effectiveness of moral messages on public health behavioral intentions during the COVID-19 pandemic.* 2020.

19. Moran, M.B. and S. Sussman, *Translating the link between social identity and health behavior into effective health communication strategies: An experimental application using antismoking advertisements.* Health communication, 2014. **29**(10): p. 1057-1066.

20. Abu-Akel, A., A. Spitz, and R. West, *The Effect of Spokesperson Attribution on Public Health Messaging During the COVID-19 Pandemic.* 2020.

21. Vaughan, E. and T. Tinker, *Effective health risk communication about pandemic influenza for vulnerable populations.* American journal of public health, 2009. **99**(S2): p. S324-S332.

22. Andreoni, J., W. Harbaugh, and L. Vesterlund, *Altruism in experiments. The new palgrave dictionary in economics*. 2008, Macmillan, New York.

23. Charness, G. and U. Gneezy, *What's in a name? Anonymity and social distance in dictator and ultimatum games.* Journal of Economic Behavior & Organization, 2008. **68**(1): p. 29-35.

24. Eckel, C.C. and P.J. Grossman, *Altruism in anonymous dictator games.* Games and economic behavior, 1996. **16**(2): p. 181-191.

25. Bohnet, I. and B.S. Frey, *Social distance and other-regarding behavior in dictator games: Comment.* American Economic Review, 1999. **89**(1): p. 335-339.

For peer review only

26. Burnham, T.C., *Engineering altruism: A theoretical and experimental investigation of anonymity and gift giving.* Journal of Economic Behavior & Organization, 2003. **50**(1): p. 133-144.

27. Jones, B. and H. Rachlin, *Social discounting.* Psychological science, 2006. **17**(4): p. 283-286.

28. Leider, S., et al., *Directed altruism and enforced reciprocity in social networks.* The Quarterly Journal of Economics, 2009. **124**(4): p. 1815-1851.

29. Goeree, J.K., A. Riedl, and A. Ule, *In search of stars: Network formation among heterogeneous agents.* Games and Economic Behavior, 2009. **67**(2): p. 445-466.

30. Stoffel, S. and B. Herrmann, *How Does Communicating Herd Immunity Affect Immunization Intentions?: Evidence From a Cross-Country Online Experiment.* International Journal of Applied Behavioral Economics (IJABE), 2021. **10**(4): p. 12-20.

31. Betsch, C., et al., *On the benefits of explaining herd immunity in vaccine advocacy.* Nature human behaviour, 2017. **1**(3): p. 1-6.

32. Kelly, M., et al., *Factors associated with vaccine intention in adults living in England who either did not want or had not yet decided to be vaccinated against COVID-19.* 2021.

33. Klett-Tammen, C.J., et al., *Determinants of tetanus, pneumococcal and influenza vaccination in the elderly: a representative cross-sectional study on knowledge, attitude and practice (KAP).* BMC public health, 2015. **16**(1): p. 1-9.

34. Loubet, P., et al., *Pneumococcal and influenza vaccine uptake in adults with inflammatory bowel disease in France: Results from a web-based study.* Digestive and Liver Disease, 2018. **50**(6): p. 563-567.

35. Stoffel, S.T., et al., *Testing the decoy effect to increase interest in colorectal cancer screening.* PloS one, 2019. **14**(3): p. e0213668.

36. Stoffel, S.T., et al., *Testing verbal quantifiers for social norms messages in cancer screening: evidence from an online experiment.* BMC public health, 2019. **19**(1): p. 1-11.

37. Betsch, C., et al., *Beyond confidence: Development of a measure assessing the 5C psychological antecedents of vaccination.* PloS one, 2018. **13**(12): p. e0208601.

38. Cohen, J., *Statistical power analysis for the behavioural sciences (2nd edn.). Hillside*. 1988, NJ: Erlbaum.

39. Thompson, H.S., et al., *Factors associated with racial/ethnic group–based medical mistrust and perspectives on COVID-19 vaccine trial participation and vaccine uptake in the US.* JAMA Network Open, 2021. **4**(5): p. e2111629-e2111629.

For peer review only

40. Sheeran, P., *Intention—behavior relations: a conceptual and empirical review.* European review of social psychology, 2002. **12**(1): p. 1-36.

41. Webb, T.L. and P. Sheeran, *Does changing behavioral intentions engender behavior change? A meta-analysis of the experimental evidence.* Psychological bulletin, 2006. **132**(2): p. 249.

42. Milkman, K.L., et al., *Using implementation intentions prompts to enhance influenza vaccination rates.* Proceedings of the National Academy of Sciences, 2011. **108**(26): p. 10415-10420.

43. Nino, M., et al., *Race and ethnicity, gender, and age on perceived threats and fear of COVID-19: Evidence from two national data sources.* SSM Popul Health, 2021. **13**: p. 100717.

For peer review only

Figure 1. Flow through experiment

For peer review only

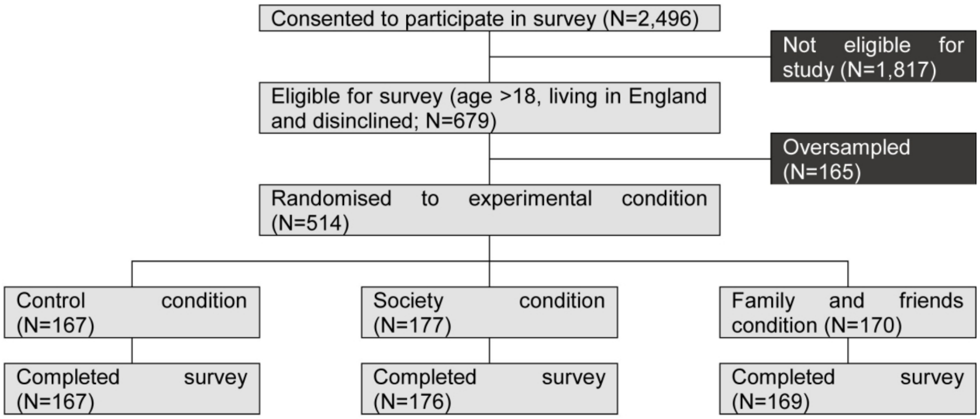

Flow through experiment

186x99mm (300 x 300 DPI)

Supplementary figures

Figure S1. Distribution of vaccination intentions across the experimental conditions

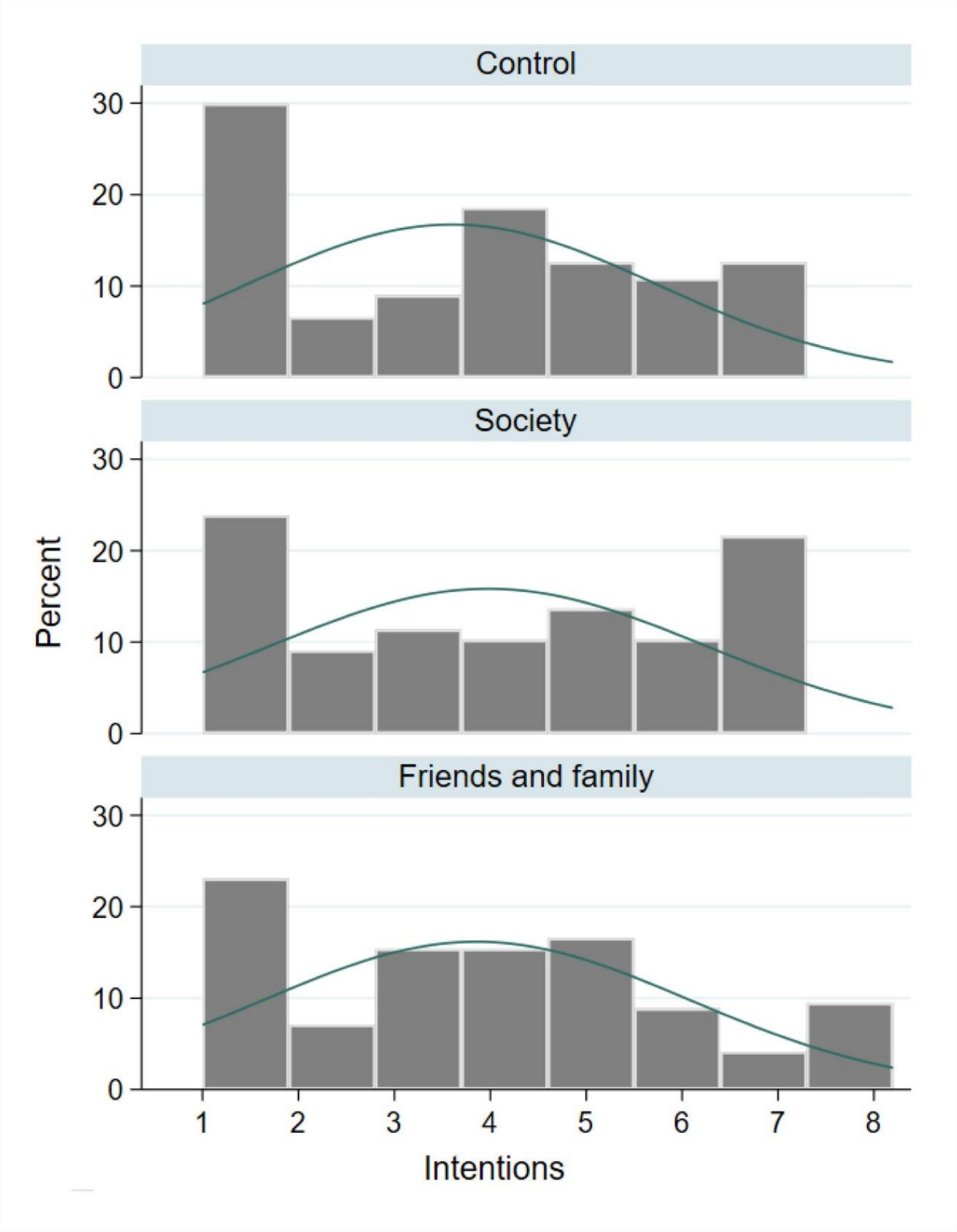

Figure S2. Distribution of perceived social importance of the vaccine across the experimental conditions

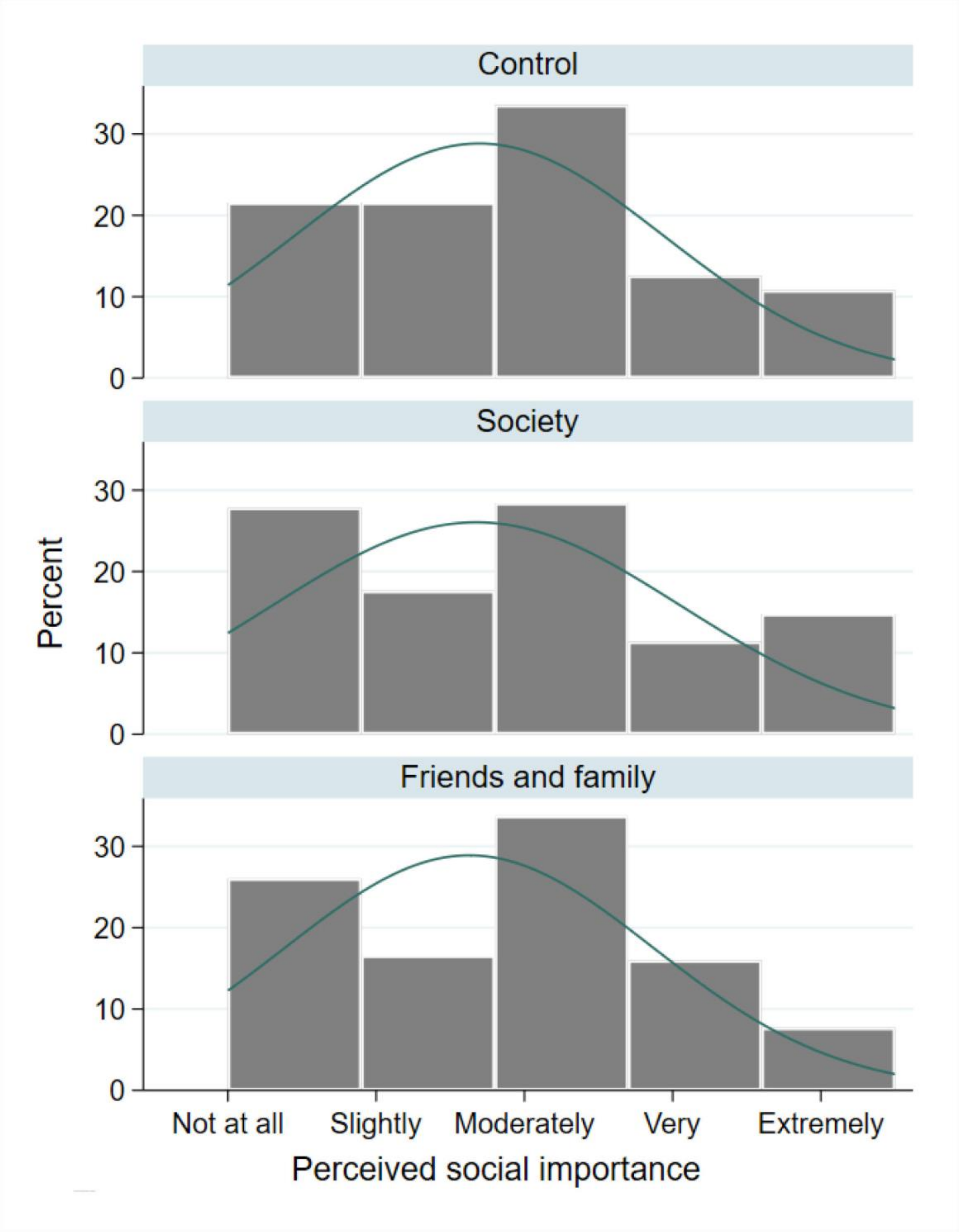

Figure S3. Distribution of the expected vaccination uptake across the experimental conditions

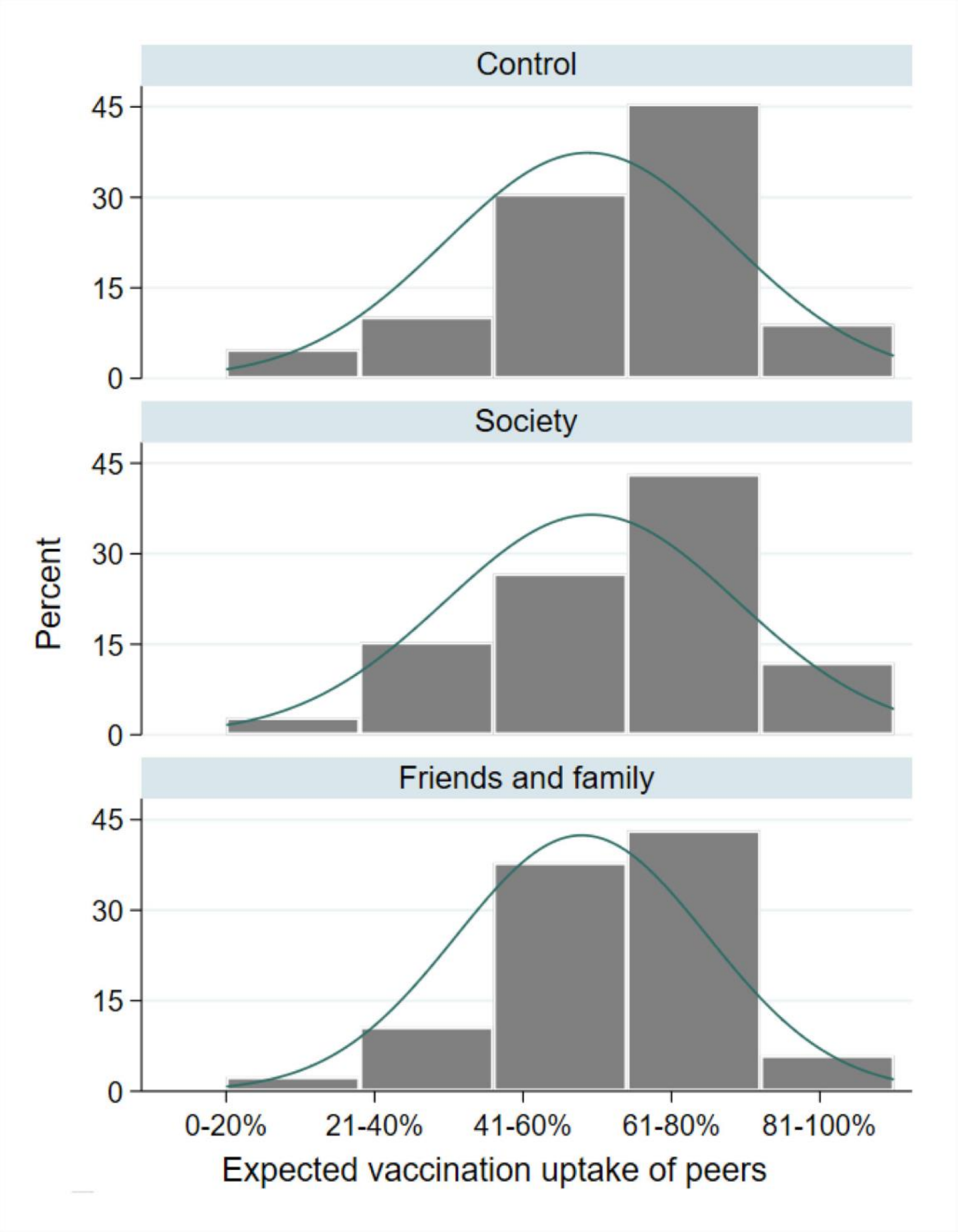

Figure S4. Attitudes towards vaccination

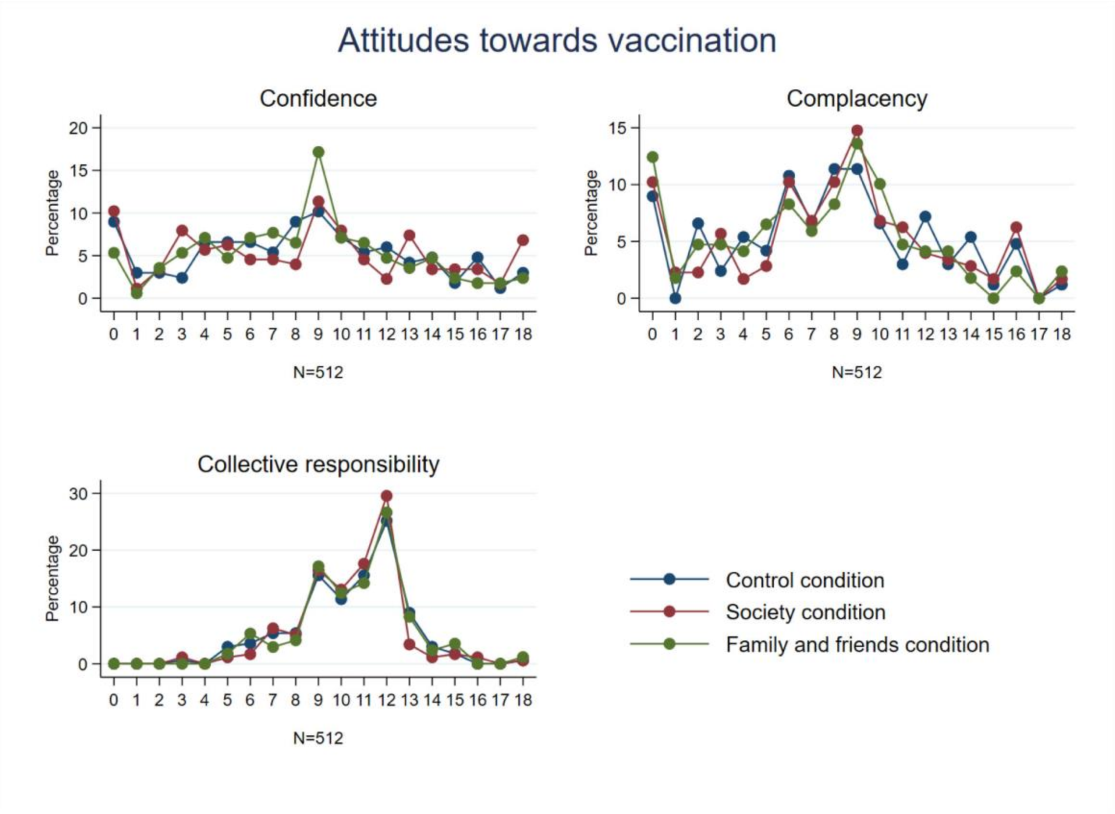

w only

Supplementary tables

Table S1: Reasons for not wanting to get vaccinated after experimental manipulation*

	Overall sample (N=306)		Only White ethnic background (N=118)		Model 3: Black, Asian, mixed or other ethnic background (N=188)	
Reason	N	(%)	N	(%)	N	(%)
Fear of side effect	201	65.9	83	70.9	118	62.8
Vaccine does not offer protection	115	37.7	47	40.2	68	36.2
Critical reports in media	69	22.6	25	21.4	44	23.4
General objections against vaccination	63	20.7	28	23.9	35	18.6
Fear of needles	44	14.4	20	17.1	24	12.8
Other reasons	37	12.1	16	13.7	21	11.2
Disease is harmless	30	9.8	8	6.8	22	11.7
Discouraged by family/friends' opinion	26	8.5	9	7.7	17	9.0
Discouraged by physician's opinion	17	5.6	9	7.7	8	4.3
Difficulties getting to the appointment	14	4.6	6	5.1	8	4.3
Too much effort	8	2.6	2	1.7	6	3.2

*only answered by study participants that stated low vaccination intentions [1-4] after the experimental manipulation

For peer review only

Table S2: Descriptive statistics across experimental conditions

	Control (N=167)		Society (N=176)		Family and friends (N=169)		Total (N=512)	
	N	(%)	N	(%)	N	(%)	N	(%)
Vaccination status								
Have been invited	101	(60.5%)	94	(53.4%)	107	(63.3%)	302	(59.0%)
Not been invited yet	66	(39.5%)	82	(46.6%)	62	(36.7%)	210	(41.0%)
Age								
18-24 years old	54	(32.3%)	52	(29.5%)	43	(25.4%)	149	(29.1%)
25-34 years old	74	(44.3%)	90	(51.1%)	72	(42.6%)	236	(46.1%)
35-44 years old	14	(8.4%)	14	(8.0%)	35	(20.7%)	63	(12.3%)
45+ years old	25	(15.0%)	20	(11.3%)	19	(11.2%)	64	(12.5%)
Gender								
Male	74	(44.3%)	81	(46.0%)	67	(39.6%)	222	(43.4%)
Female	93	(55.7%)	93	(52.8%)	99	(58.6%)	285	(55.7%)
Non-binary	0	(0.0%)	2	(1.1%)	3	(1.8%)	5	(1.0%)
Ethnicity								
White	72	(43.1%)	78	(44.3%)	57	(33.7%)	207	(40.4%)
Mixed	16	(9.6%)	30	(17.0%)	23	(13.6%)	69	(13.5%)
Asian	30	(18.0%)	34	(19.3%)	36	(21.3%)	100	(19.5%)
Black	45	(26.9%)	32	(18.2%)	46	(27.2%)	123	(24.0%)
Arab or other	4	(2.4%)	2	(1.1%)	7	(4.1%)	13	(2.5%)
A-levels								
No	29	(17.4%)	31	(17.6%)	46	(27.2%)	106	(20.7%)
Yes	138	(82.6%)	145	(82.4%)	123	(72.8%)	406	(79.3%)
Paid employment								
No	49	(29.3%)	47	(26.7%)	58	(34.3%)	154	(30.1%)
Yes	118	(70.7%)	129	(73.3%)	111	(65.7%)	358	(69.9%)
Living condition								
Alone	89	(53.3%)	91	(51.7%)	96	(56.8%)	276	(53.9%)
With someone	78	(46.7%)	85	(48.3%)	73	(43.2%)	236	(46.1%)

For peer review only

Table S3: Binary logistic regression on active interest to read more about vaccination

		Model 1: Overall sample					Model 2: Only White ethnic background					Model 3: Black, Asian, mixed or other ethnic backgound			
	(%)	OR	95% CI	aOR	95% CI	(%)	OR	95% CI	aOR	95% CI	(%)	OR	95% CI	aOR	95% CI
Condition															
Control	34.7	Ref.		Ref.		33.3	Ref.		Ref.		35.8	Ref.		Ref.	
Society	44.9	1.531	0.990 - 2.366	1.472	0.940 - 2.307	42.3	1.446	0.726 - 2.882	1.587	0.891 - 2.827	46.9	1.587	0.891 - 2.827	1.570	0.870 - 2.833
Family and friends	42.6	1.395	0.898 - 2.168	1.484	0.933 - 2.360	47.4	1.896	0.876 - 4.103	1.205	0.685 - 2.119	40.1	1.205	0.685 - 2.119	1.270	0.704 - 2.290
Vaccination status															
Invited	38.1	Ref.		Ref.		38.6	Ref.		Ref.		37.7	Ref.		Ref.	
Not invited	44.8	1.318	0.921 - 1.884	1.581	1.067 - 2.342*	42.8	1.238	0.701 - 2.185	1.456	0.770 - 2.754	45.4	1.372	0.865 - 2.176	1.621	0.979 - 2.685
Age															
18-24 years old	36.9	Ref.		Ref.		33.3	Ref.		Ref.		38.6	Ref.		Ref.	
25-34 years old	39.4	1.112	0.728 - 1.697	1.182	0.735 - 1.901	40.2	1.343	0.664 - 2.717	1.597	0.734 - 3.474	38.7	1.002	0.581 - 1.727	0.901	0.489 - 1.662
35-44 years old	50.8	1.764	0.973 - 3.200	1.722	0.889 - 3.333	53.8	2.333	0.672 - 8.101	2.726	0.707 - 10.503	50.0	1.590	0.802 - 3.150	1.501	0.693 - 3.252
45+ years old	45.3	1.416	0.782 - 2.565	1.915	0.986 - 3.718	48.3	1.867	0.726 - 4.796	2.415	0.839 - 6.951	42.9	1.192	0.546 - 2.601	1.351	0.559 - 3.266
Gender															
Male	42.3	Ref.		Ref.		39.8	Ref.		Ref.		44.5	Ref.		Ref.	
Female	39.6	0.895	0.626 - 1.278	0.898	0.617 - 1.308	41.6	1.076	0.616 - 1.882	1.113	0.614 - 2.019	38.6	0.782	0.490 - 1.249	0.809	0.494 - 1.325
Non-binary	40.0	0.908	0.149 - 5.541	0.657	0.102 - 4.213	33.3	0.756	0.066 - 8.611	0.547	0.045 - 6.582	50.0	1.245	0.076 - 20.382	1.112	0.065 - 19.070
Ethnicity															
White	40.6	Ref.		Ref.					.			.		.	
Mixed	40.6	1.000	0.574 - 1.742	0.908	0.510 - 1.616										
Asian	52.0	1.586	0.981 - 2.565	1.468	0.884 - 2.440										
Black	34.9	0.787	0.495 - 1.250	0.742	0.456 - 1.209										
Arab or other	15.4	0.266	0.058 - 1.232	0.280	0.059 - 1.323										
A-levels															
No	33.0	Ref.		Ref.		32.7	Ref.		Ref.		33.3	Ref.		Ref.	
Yes	42.9	1.521	0.970 - 2.386	1.692	1.042 - 2.746*	43.2	1.568	0.809 - 3.035	2.253	1.072 - 4.735*	42.6	1.486	0.801 - 2.759	1.611	0.827 - 3.140
Living condition															
Alone	39.1	Ref.		Ref.		41.1	Ref.		Ref.		38.1	Ref.		Ref.	
With somebody	42.8	1.164	0.817 - 1.657	0.973	0.652 - 1.452	40.2	0.964	0.553 - 1.683	0.873	0.476 - 1.603	45.2	1.337	0.841 - 2.125	1.196	0.700 - 2.041
Paid employment															
No	38.3	Ref.		Ref.		49.1	Ref.		Ref.		32.0	Ref.		Ref.	
Yes	41.9	1.161	0.789 - 1.710	1.107	0.727 - 1.686	37.3	0.617	0.333 - 1.142	0.606	0.310 - 1.183	45.5	1.756	1.058 - 2.914*	1.627	0.947 - 2.795
N		512		512			207		207			305		305	

* $p<0.05$; ** $p<0.01$

For peer review only

Table S4: Ordered logistic regression on perceived importance of vaccination [1;5]

	Model 1: Overall sample				Model 2: Only White ethnic background				Model 3: Black, Asian, mixed or other ethnic backgound			
	OR	95% CI	aOR	95% CI	OR	95% CI	aOR	95% CI	OR	95% CI	aOR	95% CI
Condition												
Control	Ref.		Ref.		Ref.		Ref.		Ref.		Ref.	
Society	0.948	0.649 - 1.386	0.897	0.608 - 1.325	1.493	0.836 - 2.668	1.458	0.794 - 2.677	0.647	0.391 - 1.072	0.627	0.376 - 1.047
Family and friends	0.931	0.639 - 1.357	1.042	0.706 - 1.539	1.271	0.692 - 2.334	1.833	0.961 - 3.496	0.751	0.462 - 1.222	0.829	0.502 - 1.369
Vaccination status												
Invited	Ref.		Ref.		Ref.		Ref.		Ref.		Ref.	
Not invited	1.317	0.964 - 1.799	1.200	0.859 - 1.677	0.919	0.562 - 1.503	0.563	0.321 - 0.990*	1.728	1.149 - 2.599**	1.774	1.151 - 2.733**
Age												
18-24 years old	Ref.		Ref.		Ref.		Ref.		Ref.		Ref.	
25-34 years old	0.851	0.592 - 1.224	0.813	0.547 - 1.208	0.953	0.527 - 1.724	0.754	0.397 - 1.432	0.703	0.439 - 1.126	0.690	0.411 - 1.159
35-44 years old	0.754	0.451 - 1.260	0.689	0.393 - 1.208	0.495	0.180 - 1.362	0.364	0.119 - 1.112	0.905	0.491 - 1.669	0.905	0.463 - 1.769
45+ years old	0.401	0.238 - 0.675**	0.428	0.240 - 0.761**	0.321	0.143 - 0.719**	0.265	0.105 - 0.665**	0.478	0.237 - 0.965*	0.501	0.230 - 1.094
Gender												
Male	Ref.		Ref.		Ref.		Ref.		Ref.		Ref.	
Female	0.557	0.405 - 0.765**	0.537	0.387 - 0.745**	0.411	0.249 - 0.680**	0.375	0.223 - 0.631**	0.715	0.472 - 1.085	0.707	0.459 - 1.090
Non-binary	0.552	0.130 - 2.341	0.509	0.118 - 2.205	0.857	0.154 - 4.754	1.124	0.182 - 6.955	0.163	0.014 - 1.941	0.153	0.012 - 1.891
Ethnicity												
White	Ref.		Ref.				.		.		.	
Mixed	0.701	0.433 - 1.137	0.684	0.416 - 1.125								
Asian	1.132	0.740 - 1.731	1.100	0.707 - 1.710								
Black	0.718	0.482 - 1.071	0.633	0.417 - 0.960*								
Arab or other	0.693	0.259 - 1.854	0.707	0.253 - 1.975								
A-levels												
No	Ref.		Ref.		Ref.		Ref.		Ref.		Ref.	
Yes	1.741	1.188 - 2.549**	1.793	1.190 - 2.701**	2.221	1.277 - 3.865**	2.301	1.260 - 4.204**	1.435	0.840 - 2.453	1.466	0.827 - 2.599
Living condition												
Alone	Ref.		Ref.		Ref.		Ref.		Ref.		Ref.	
With somebody	1.036	0.758 - 1.416	1.088	0.770 - 1.536	0.908	0.558 - 1.478	0.811	0.483 - 1.360	1.117	0.742 - 1.683	1.415	0.883 - 2.266
Paid employment												
No	Ref.		Ref.		Ref.		Ref.		Ref.		Ref.	
Yes	1.263	0.906 - 1.761	1.120	0.788 - 1.590	1.545	0.910 - 2.625	1.252	0.714 - 2.196	1.093	0.711 - 1.679	1.076	0.680 - 1.702
N	512		512		207		207		305		305	

* *p*<0.05; ** *p*<0.01

For peer review only

Table S5: Ordered logistic regression on expected uptake of vaccination [1;5]

	Model 1: Overall sample				Model 2: Only White ethnic background				Model 3: Black, Asian, mixed or other ethnic backgound			
	OR	95% CI	aOR	95% CI	OR	95% CI	aOR	95% CI	OR	95% CI	aOR	95% CI
Condition												
Control	Ref.		Ref.		Ref.		Ref.		Ref.		Ref.	
Society	1.036	0.696 - 1.540	1.048	0.700 - 1.571	1.260	0.694 - 2.288	1.443	0.779 - 2.674	0.856	0.501 - 1.461	0.863	0.504 - 1.480
Family and friends	0.864	0.585 - 1.276	0.885	0.592 - 1.324	0.852	0.443 - 1.636	1.000	0.508 - 1.970	0.874	0.535 - 1.429	0.823	0.496 - 1.365
Vaccination status												
Invited	Ref.		Ref.		Ref.		Ref.		Ref.		Ref.	
Not invited	0.598	0.431 - 0.830**	0.640	0.451 - 0.908*	0.491	0.290 - 0.831**	0.513	0.290 - 0.909*	0.675	0.443 - 1.029	0.720	0.459 - 1.129
Age												
18-24 years old	Ref.		Ref.		Ref.		Ref.		Ref.		Ref.	
25-34 years old	1.118	0.765 - 1.635	1.017	0.666 - 1.553	1.375	0.734 - 2.574	1.106	0.562 - 2.179	0.915	0.561 - 1.493	0.988	0.573 - 1.702
35-44 years old	1.405	0.818 - 2.415	1.324	0.737 - 2.380	1.283	0.399 - 4.120	1.431	0.444 - 4.608	1.442	0.778 - 2.673	1.486	0.748 - 2.954
45+ years old	1.472	0.849 - 2.553	1.332	0.727 - 2.441	1.479	0.622 - 3.517	1.197	0.464 - 3.088	1.463	0.708 - 3.020	1.557	0.694 - 3.493
Gender												
Male	Ref.		Ref.		Ref.		Ref.		Ref.		Ref.	
Female	0.839	0.606 - 1.162	0.802	0.573 - 1.123	0.840	0.503 - 1.402	0.838	0.495 - 1.418	0.875	0.570 - 1.345	0.830	0.533 - 1.292
Non-binary	0.256	0.056 - 1.171	0.289	0.060 - 1.401	0.170	0.021 - 1.342	0.231	0.029 - 1.867	0.406	0.048 - 3.450	0.469	0.050 - 4.374
Ethnicity												
White	Ref.		Ref.				.		.		.	
Mixed	0.839	0.507 - 1.390	0.842	0.502 - 1.412								
Asian	1.067	0.686 - 1.659	1.035	0.649 - 1.650								
Black	0.653	0.432 - 0.989*	0.653	0.425 - 1.005								
Arab or other	0.838	0.311 - 2.258	0.896	0.324 - 2.480								
A-levels												
No	Ref.		Ref.		Ref.		Ref.		Ref.		Ref.	
Yes	1.312	0.873 - 1.973	1.272	0.827 - 1.958	1.408	0.771 - 2.571	1.249	0.661 - 2.362	1.306	0.744 - 2.292	1.363	0.741 - 2.505
Living condition												
Alone	Ref.		Ref.		Ref.		Ref.		Ref.		Ref.	
With somebody	0.995	0.722 - 1.370	0.828	0.581 - 1.179	1.019	0.612 - 1.696	0.858	0.501 - 1.469	0.942	0.620 - 1.432	0.852	0.533 - 1.362
Paid employment												
No	Ref.		Ref.		Ref.		Ref.		Ref.		Ref.	
Yes	1.158	0.815 - 1.644	1.102	0.761 - 1.596	1.686	0.950 - 2.990	1.555	0.859 - 2.812	0.914	0.585 - 1.427	0.876	0.545 - 1.408
N	512		512		207		207		305		305	

* $p<0.05$; ** $p<0.01$

For peer review only

Table S6. Adjusted logistic regression with interaction terms for condition and ethnicity on intentions, expectations and perceived social importance and active interest (N=512)

	Model 1: Intentions [1;8]		Model 2: Expectations [1;5]		Model 3: Social importance [1;5]		Model 4: Active interest [0;1]	
	aOR	95% CI	aOR	95% CI	aOR	95% CI	aOR	95% CI
Condition and ethnicity								
Control – White	Ref.		Ref.		Ref.		Ref.	
Control – Ethnic minoties	1.304	0.747 - 2.277	1.019	0.570 - 1.824	1.264	0.721 - 2.215	1.041	0.536 - 2.020
Society – White	1.984	1.090 - 3.614*	1.411	0.776 - 2.567	1.529	0.826 - 2.829	1.440	0.730 - 2.839
Society – Ethnic minorities	1.301	0.748 - 2.265	0.873	0.485 - 1.574	0.842	0.480 - 1.476	1.661	0.870 - 3.172
Family and friends - White	1.681	0.886 - 3.189	0.964	0.501 - 1.857	1.568	0.825 - 2.981	1.931	0.921 - 4.047
Family and friends – Ethnic minority	1.419	0.818 - 2.462	0.836	0.476 - 1.465	1.026	0.589 - 1.787	1.287	0.673 - 2.460
Vaccination status								
Invited	Ref.		Ref.		Ref.		Ref.	
Not invited	1.135	0.819 - 1.574	0.625	0.440 - 0.887**	1.164	0.833 - 1.627	1.515	1.026 - 2.237*
Age								
18-24 years old	Ref.		Ref.		Ref.		Ref.	
25-34 years old	0.951	0.647 - 1.397	1.025	0.674 - 1.559	0.804	0.543 - 1.191	1.152	0.721 - 1.840
35-44 years old	0.844	0.490 - 1.454	1.404	0.784 - 2.514	0.749	0.431 - 1.302	1.918	0.999 - 3.682
45+ years old	0.293	0.164 - 0.523**	1.346	0.737 - 2.460	0.428	0.241 - 0.761**	1.824	0.943 - 3.526
Gender								
Male	Ref.		Ref.		Ref.		Ref.	
Female	0.677	0.491 - 0.933*	0.829	0.593 - 1.158	0.546	0.394 - 0.757**	0.908	0.626 - 1.317
Non-binary	0.360	0.082 - 1.577	0.281	0.059 - 1.340	0.457	0.107 - 1.951	0.715	0.112 - 4.552
A-levels								
No	Ref.		Ref.		Ref.		Ref.	
Yes	1.629	1.079 - 2.458*	1.264	0.820 - 1.948	1.822	1.210 - 2.745**	1.783	1.097 - 2.899*
Living condition								
Alone	Ref.		Ref.		Ref.		Ref.	
With somebody	1.263	0.902 - 1.768	0.856	0.602 - 1.216	1.127	0.800 - 1.588	1.012	0.682 - 1.503
Paid employment								
No	Ref.		Ref.		Ref.		Ref.	
Yes	1.145	0.809 - 1.619	1.088	0.753 - 1.572	1.112	0.784 - 1.578	1.101	0.728 - 1.666
N	512		512		512		512	

* *p*<0.05; ** *p*<0.01

For peer review only

Page 1:

Title of Project: The effect of communicating herd immunity on vaccination intention

This study has been approved by xxxx

We would like to invite you to take part in this study. You should only take part if you want to. Before you decide whether to take part, it is important for you to read the following information carefully and discuss it with others if you wish. Please ask us if anything is unclear or if you would like more information.

What is the purpose of the study?
This study is concerned with how individuals make choices related to the coronavirus vaccination. To explore this further, we have developed a survey to ask people about their preferences.

Why have I been invited?

We are inviting adults (men and women aged 18-65) living in England to take part in this survey.

Do I have to take part?
It is up to you to decide to join the study. If you decide to withdraw you will be asked what you wish to happen to the data you have provided up that point.

What will I have to do if I take part?
If you choose to take part we would ask you to complete an online survey which should take around 10 minutes to complete.

What are the possible disadvantages and risks of taking part?
There are no physical risks for the participants. This study carries very little risk for the participants. There are no right or wrong answers to the questions. As we are asking you about health issues that may be sensitive, there is a slight possibility that you will feel uncomfortable.

What are the possible benefits of taking part?
In addition to the remuneration you receive from Qualtrics, this study will help us to better understand people's attitudes towards coronavirus vaccine.

What if there is a problem?
If you have a concern about any aspects of the study you should in the first instance contact XXX who will do her best to answer your questions. If you would like to discuss a problem with someone not directly involved in the project, you can contact the chairs of xxx research ethics committees (XXX).

Will my taking part in this project be kept confidential?
Yes. All information which is collected about you during the course of the research is anonymous and not linked to any of your personal details. This means that you cannot be recognised from the information you provide. Our procedures for storage, processing, handling and destroying the information you give us are in line with Data

For peer review only

Protection Act (1998) and the General Data Protection Regulation (2016). The survey will be conducted using SurveyMonkey which stores data collected on servers located in the United States.

What will happen to the results of the research project?
We hope to report the findings from the study in an academic journal. You will not be identified in any reports or publications from the study.

Who is organising the research?
This work is being delivered by the National Institute for Health Research Policy Research Unit in Behavioural Science. The Unit is a collaboration between Newcastle University, University College London, University of Warwick, and the London School of Hygiene and Tropical Medicine. The work was developed in collaboration with Public Health England.

Contact for further information
Researcher: XXX
Principal Researcher: XXX
UCL Data Protection Officer: XXX

Thank you for reading this information sheet and for considering to take part in this research study.

[continue page 2]

For peer review only

Consent form

Thank you for considering taking part in this research. If you have any questions arising from the Information explanation already given to you, please ask the researcher before you decide whether to join in.

I confirm that I understand that by ticking/initialling each box below I am consenting to this element of the study. I understand that it will be assumed that unticked/uninitialled boxes means that I DO NOT consent to that part of the study. I understand that by not giving consent for any one element that I may be deemed ineligible for the study.

	Tick Box
**1. I confirm that I have read and understood the information sheet for the study above. I have had an opportunity to consider the information and what will be expected of me. I have also had the opportunity to ask questions.**	
**2. I understand that my participation is voluntary and that I am free to withdraw at any time without giving a reason**	
**3. I understand that my data gathered in this study will be stored anonymously and securely. It will not be possible to identify me in any publications.**	
**4. I understand that the data will not be made available to any commercial organisations but is solely the responsibility of the researchers undertaking this study.**	
**5. I am aware of who I should contact if I wish to lodge a complaint.**	
**6. I voluntarily agree to take part in this study.**	
**7. I understand that information collected in this survey is stored on servers based in the USA.**	

[continue page 3]

For peer review only

Page 3: First, we want to make sure you're a good fit for our study. Please answer the questions below so we can check.

1. How old are you in years?
   - 17 or younger [filter out]
   - 18-24 years old
   - 25-34 years old
   - 35-44 years old
   - 55-54 years old
   - 55 or older

2. What is your gender?
   - Female
   - Male
   - Other

3. Where do you live?
   - Scotland [filter out]
   - Northern Ireland [filter out]
   - Wales [filter out]
   - North East
   - North West
   - Yorkshire and the Humber
   - West Midlands
   - East Midlands
   - South West
   - South East
   - East of England
   - Greater London

4. Which of the following describes your ethnic group?
   - White British [filter out]
   - Other White background [filter out]
   - Black background
   - Asian background
   - Mixed background
   - Any other

5. Have you already been invited for a coronavirus vaccine?
   - Yes [filter out]
   - No

6. Do you or someone in your household have a health condition, is pregnant or age 70+? (question adapted from NIHR questionnaire 2020)
   - Yes
   - No

For peer review only

7. Which of the following numbers represents the biggest risk of getting a disease? (question from Lipkus et al., 2001, used in Stoffel et al., 2019)

- 1/10
- 1/100
- 1/1000
- Don't know

[continue page 4]

For peer review only

Page 4:
Thank you for filling out the initial questions. We would now like to introduce the main part of the survey.

The NHS is offering free coronavirus vaccine to people living in England.

8. Please evaluate how much you disagree or agree with the following statement.

When it's available to me, I will have a coronavirus vaccine. (question adapted from YouGov questionnaire)

- Strongly disagree [continue page 5/6/7]
- Disagree [continue page 5/6/7]
- Neither agree nor disagree [continue page 5/6/7]
- Agree [filter out]
- Strongly agree [filter out]

For peer review only

Page 5: Control condition (33.3%):

Imagine that you have received the following invitation letter from the NHS.

NHS | HM Government

CORONAVIRUS VACCINATION

We are writing to invite you to get a free coronavirus vaccine.

This is part of a national programme that will involve millions of people receiving this vaccine. Each person who gets the vaccine will need to receive two separate doses.

In order to get the vaccine, you will need to go to your registered GP. The two doses will be given to you on two separate visits

Get vaccinated to protect you from Covid-19.

9a. 1. Would you get vaccinated against the coronavirus? (1 means definitely not while 8 indicates definitely yes) (question adapted from unpublished study by Stoffel & Herrmann)

- 1
- 2
- 3
- 4
- 5
- 6
- 7
- 8

10a. Do you think that most people will get vaccinated? (question adapted from unpublished study by Stoffel & Herrmann)

- No
- Yes

11a. Do you believe that getting vaccinated against the virus is socially important? (question adapted from unpublished study by Stoffel & Herrmann)

- No
- Yes

[continue page 8]

For peer review only

Page 6: Society condition (33.3%):

Imagine that you have received the following invitation letter from the NHS.

NHS | HM Government

CORONAVIRUS VACCINATION

We are writing to invite you to get a free coronavirus vaccine.

This is part of a national programme that will involve millions of people receiving this vaccine. Each person who gets the vaccine will need to receive two separate doses.

In order to get the vaccine, you will need to go to your registered GP. The two doses will be given to you on two separate visits

Get vaccinated to protect you and the vulnerable people in Great Britain from Covid-19.

9b. 1. Would you get vaccinated against the coronavirus? (1 means definitely not while 8 indicates definitely yes) (question adapted from unpublished study by Stoffel & Herrmann)

- 1
- 2
- 3
- 4
- 5
- 6
- 7
- 8

10b. Do you think that most people will get vaccinated? (question adapted from unpublished study by Stoffel & Herrmann)

- No
- Yes

11b. Do you believe that getting vaccinated against the virus is socially important? (question adapted from unpublished study by Stoffel & Herrmann)

- No
- Yes

[continue page 8]

Page 7: Friends condition (33.3%):

Imagine that you have received the following invitation letter from the NHS.

NHS | HM Government

CORONAVIRUS VACCINATION

We are writing to invite you to get a free coronavirus vaccine.

This is part of a national programme that will involve millions of people receiving this vaccine. Each person who gets the vaccine will need to receive two separate doses.

In order to get the vaccine, you will need to go to your registered GP. The two doses will be given to you on two separate visits

Get vaccinated to protect you and your vulnerable friends and family members, such as your children, your parents or your grandparents from Covid-19.

9c. 1. Would you get vaccinated against the coronavirus? (1 means definitely not while 8 indicates definitely yes) (question adapted from unpublished study by Stoffel & Herrmann)

- 1
- 2
- 3
- 4
- 5
- 6
- 7
- 8

10c. Do you think that most people will get vaccinated? (question adapted from unpublished study by Stoffel & Herrmann)

- No
- Yes

11c. Do you believe that getting vaccinated against the virus is socially important? (question adapted from unpublished study by Stoffel & Herrmann)

- No
- Yes

[continue page 8]

Page 8: Attitudes towards vaccination (taken from Betsch et al., 2018)

12. Please indicate how strongly you agree or disagree with all the following statements

Item	Response options						
	**strongly disagree**	**moderately disagree**	**slightly disagree**	**neutral**	**slightly agree**	**moderately agree**	**Strongly agree**
I am completely confident that vaccines are safe.							
Vaccinations are effective.							
Regarding vaccines, I am confident that public authorities decide in the best interest of the community.							
Everyday stress prevents me from getting vaccinated.							
For me, it is inconvenient to receive vaccinations.							
Visiting the doctor's makes me feel uncomfortable; this keeps me from getting vaccinated.							
When I think about getting vaccinated, I weigh benefits and risks to make the best decision possible.							
For each and every vaccination, I closely consider whether it is useful for me.							
It is important for me to fully understand the topic of vaccination, before I get vaccinated.							

[continue page 8]

For peer review only

Page 9. Additional information

13. Before continuing with the survey, would you be interested in reading some more facts and figures about the vaccination appointment on the next page or do you prefer to skip it? (question adapted from Stoffel et al., 2019)

- Read information on next page before continuing with survey [continue page 10]
- Skip information on next page and continue with survey [continue page 12]

Page 10: Information about the vaccination process.

How the coronavirus vaccine is given

The coronavirus vaccine should be administered by intramuscular injection into the deltoid muscle of the upper arm.

It's given as 2 doses. You will have the 2nd dose 3 to 12 weeks after having the 1st dose.

How safe is the coronavirus vaccine?

The vaccines approved for use in the UK have met strict standards of safety, quality and effectiveness set out by the independent Medicines and Healthcare products Regulatory Agency (MHRA).

Any coronavirus vaccine that is approved must go through all the clinical trials and safety checks all other licensed medicines go through. The MHRA follows international standards of safety.

Other vaccines are being developed. They will only be available on the NHS once they have been thoroughly tested to make sure they are safe and effective.

So far, thousands of people have been given a coronavirus vaccine and reports of serious side effects, such as allergic reactions, have been very rare. No long-term complications have been reported.

Coronavirus vaccine side effects

Most side effects of the coronavirus vaccine, if you do experience any, are mild and should not last longer than a week, such as:

- a sore arm where the needle went in
- feeling tired
- a headache
- feeling achy
- feeling or being sick

You can take painkillers, such as paracetamol, if you need to.

If you have a high temperature you may have coronavirus or another infection.

For peer review only

As with any symptoms, if they get worse or you are worried, call 111.

Allergic reactions

Tell healthcare staff before you are vaccinated if you've ever had an unexplained anaphylaxis or a serious allergic reaction (anaphylaxis) due to food, an identified drug or vaccine, multiple classes of drugs, or an insect sting.

You should not have the vaccine if you've ever had a serious allergic reaction to:

- a previous vaccine
- a previous dose of the same COVID-19 vaccine
- some medicines, household products or cosmetics (especially those which contain polyethylene glycol (PEG) or any of the other ingredients of the vaccine(s) (PHE, 2021))

Serious allergic reactions are very rare. If you do have a reaction to the vaccine, it usually happens in minutes. Staff giving the vaccine are trained to deal with allergic reactions and treat them immediately.

[continue page 11]

Page 11: Comprehension questions on the additional information

14a. When would you be given the second dose of the vaccine
- 1-2 weeks after the first one
- 3-12 weeks after the first one
- 13-20 weeks after the first one

14b. Is high temperature a common side effect of the vaccine?
- Yes
- No

14c. Are you allowed to take painkillers in case you suffer from side effects?
- Yes
- No

14d. When would allergic reaction to the vaccine usually happen?
- In seconds
- In minutes
- In hours

[continue page 12]

For peer review only

Page 12: About yourself (question adapted from Stoffel et al., 2019)

15. What is the highest educational qualification you have?
    - no qualifications
    - GCSE level
    - A-level
    - higher education

16. Are you currently in paid employment?
    - Yes
    - No

17. What is your marital status?
    - Single
    - Married or living with partner
    - Divorced, separated or widowed

[continue page 13]

Page 13: End of the survey

Thank you for your participation!
The information collected from this survey will be used to examine attitudes towards coronavirus vaccination. The aim of this study is to investigate the role of the appointment characteristics on vaccination intentions among adults (men and women aged 18-50) with low interest. If you would like more information about the study, please contact XXX.

[redirect to the survey vendors website according to provided link]

For peer review only

References

Betsch, C., Schmid, P., Heinemeier, D., Korn, L., Holtmann, C., & Böhm, R. (2018). Beyond confidence: Development of a measure assessing the 5C psychological antecedents of vaccination. PloS one, 13(12), e0208601. https://doi.org/10.1371/journal.pone.0208601

Lipkus I.M., Samsa G., Rimer B.K. (2001). General performance on a numeracy scale among highly educated samples. Medical Decision Making, 21(1), 37–44. https://doi.org/10.1177/0272989X0102100105 PMID: 11206945

Public Health England (PHE). (2021) COVID-19: the green book, chapter 14a (*Last updated 26 January 2021*). *Available at PHE gateway number 2020300. https://www.gov.uk/government/publications/covid-19-the-green-book-chapter-14a*

Stoffel, S., & Herrmann, B. (2021). How Does Communicating Herd Immunity Affect Immunization Intentions?: Evidence From a Cross-Country Online Experiment. International Journal of Applied Behavioral Economics (IJABE), 10(4), 12-20.

Stoffel, S. T., Yang, J., Vlaev, I., & von Wagner, C. (2019). Testing the decoy effect to increase interest in colorectal cancer screening. PloS one, 14(3), e0213668.

For peer review only
